# Transcriptional Profiles of Mating-Responsive Genes from Testes and Male Accessory Glands of the Mediterranean Fruit Fly, *Ceratitis capitata*

**DOI:** 10.1371/journal.pone.0046812

**Published:** 2012-10-11

**Authors:** Francesca Scolari, Ludvik M. Gomulski, José M. C. Ribeiro, Paolo Siciliano, Alice Meraldi, Marco Falchetto, Angelica Bonomi, Mosè Manni, Paolo Gabrieli, Alberto Malovini, Riccardo Bellazzi, Serap Aksoy, Giuliano Gasperi, Anna R. Malacrida

**Affiliations:** 1 Department of Biology and Biotechnology, University of Pavia, Pavia, Italy; 2 Section of Vector Biology, Laboratory of Malaria and Vector Research, National Institute of Allergy and Infectious Diseases, Rockville, Maryland, United States of America; 3 IRCCS, Fondazione Salvatore Maugeri, Pavia, Italy; 4 Istituto Universitario di Studi Superiori (IUSS), Pavia, Italy; 5 Department of Industrial and Information Engineering, University of Pavia, Pavia, Italy; 6 Department of Epidemiology of Microbial Diseases, Yale School of Public Health, New Haven, Connecticut, United States of America; University of Kentucky, United States of America

## Abstract

**Background:**

Insect seminal fluid is a complex mixture of proteins, carbohydrates and lipids, produced in the male reproductive tract. This seminal fluid is transferred together with the spermatozoa during mating and induces post-mating changes in the female. Molecular characterization of seminal fluid proteins in the Mediterranean fruit fly, *Ceratitis capitata*, is limited, although studies suggest that some of these proteins are biologically active.

**Methodology/Principal Findings:**

We report on the functional annotation of 5914 high quality expressed sequence tags (ESTs) from the testes and male accessory glands, to identify transcripts encoding putative secreted peptides that might elicit post-mating responses in females. The ESTs were assembled into 3344 contigs, of which over 33% produced no hits against the nr database, and thus may represent novel or rapidly evolving sequences. Extraction of the coding sequences resulted in a total of 3371 putative peptides. The annotated dataset is available as a hyperlinked spreadsheet. Four hundred peptides were identified with putative secretory activity, including odorant binding proteins, protease inhibitor domain-containing peptides, antigen 5 proteins, mucins, and immunity-related sequences. Quantitative RT-PCR-based analyses of a subset of putative secretory protein-encoding transcripts from accessory glands indicated changes in their abundance after one or more copulations when compared to virgin males of the same age. These changes in abundance, particularly evident after the third mating, may be related to the requirement to replenish proteins to be transferred to the female.

**Conclusions/Significance:**

We have developed the first large-scale dataset for novel studies on functions and processes associated with the reproductive biology of *Ceratitis capitata*. The identified genes may help study genome evolution, in light of the high adaptive potential of the medfly. In addition, studies of male recovery dynamics in terms of accessory gland gene expression profiles and correlated remating inhibition mechanisms may permit the improvement of pest management approaches.

## Introduction

In insects, the seminal fluid that conveys the spermatozoa is a complex mixture of proteins, inorganic solutes, carbohydrates and lipids produced in the male reproductive tract and transferred to the female during mating [1]–[7]. In many species these components are responsible for a complex set of physiological and behavioural changes in the female, including a reduction of receptivity to remating, increased ovulation and egg-laying, and variations in feeding activities [3], [7], [8]. The molecular and physiological functions of these substances have been most extensively investigated in *Drosophila melanogaster* [9], and particular attention has been given to seminal fluid proteins (SFPs) produced in the male accessory gland (MAGs), due to their involvement in the modulation of female post-mating responses. These accessory gland proteins (Acps) consist mainly of putative proteolysis regulators (proteases and protease inhibitors), lipid modifiers (lipases), sperm-binding candidates (Cysteine-RIch Secretory Proteins, CRISPs), antioxidants, carbohydrate-binding proteins (lectins), and many other small peptides and prohormones [10], [11].

In recent years, with the aid of next generation sequencing technologies and proteomic approaches, comprehensive studies aimed at the identification and analyses of seminal fluid proteins have been initiated in some insect species, such as the beetle *Tribolium castaneum* [12], [13], *Heliconius* butterflies [14], the honeybee *Apis mellifera* [15], the ant *Leptothorax gredleri* [16], the sandfly *Lutzomyia longipalpis* [17], the mosquitoes *Aedes aegypti* [6], [18] and *Anopheles gambiae* [19], and other *Drosophila* species [11], [20].

This is not the case for the Mediterranean fruit fly (medfly), *Ceratitis capitata*. The medfly is a tephritid pest with a worldwide geographical distribution and a history of rapid and devastating outbreaks [21], [22]. This species is the most thoroughly studied fruit fly pest at the genetic and molecular levels and has become a model for analysis of insect invasions. A recent gene discovery project provided the first major dataset of over 9,600 putative transcripts expressed in the embryo and the adult head [23]. Nevertheless, these sequences represent only a portion of the medfly transcriptome and, given the absence of a sequenced genome, this gap of knowledge is a barrier to rapid progress in every field of medfly biology.

Although several aspects of medfly reproductive biology have been widely investigated and much is known about its demography, population genetics, ecology and physiology [22], [24], [25], systematic studies of gene expression are scarce [23], [26]–[28] and characterization of seminal fluid proteins at the molecular level is still patchy [29]. Several studies suggest that the medfly possesses biologically active Acps, since males deprived of testes are still able to reduce female receptivity to further matings [30]–[32]. Moreover, it has been shown that virgin females injected with male accessory gland extracts, like mated females, shift their attention to fruit and increase their oviposition rate [33]. Other studies suggest that the medfly may possess a homologue of the most significant *Drosophila* Acp, Acp70 [34], also known as ‘sex peptide’ due to its ability to stimulate both short and long-term post-mating responses [35], [36]. In *Drosophila*, Acp70 is able to bind sperm, which functions as a sex peptide carrier [37].

The medfly male accessory glands comprise two long tubular mesodermally-derived glands, and a set of short ectodermal glands [38]. These two gland types have been shown to produce different secretions, namely lipids, polysaccharides and proteins in the long tubular, and mainly proteinaceous secretions in the short glands. Mating induces a progressive reduction in the amount of proteins in the glands, suggesting their transfer to the female during copulation [38].

Molecular and functional information on the proteins secreted in the medfly male reproductive apparatus are limited. Several genes expressed in the male accessory glands of the medfly have been identified [29], but only modest progress has been made towards an exhaustive screening of SFPs and analyses of their functions. Often, the genes encoding SFPs are difficult to identify due to their rapid evolution. Indeed, in *D. melanogaster*, the evolutionary rate of many of these genes is so fast that they lack detectable orthologues even in other *Drosophila* species [2], [39]–[42]. These SFPs are involved in the establishment of barriers to fertilization that can lead to speciation since they contribute to sperm activation, gamete interactions and ovulation. Post-copulatory competition for egg fertilization may lead to fast co-evolution between seminal proteins and proteins encoded within the female reproductive tract [43]. Independent episodes of such rapid co-evolution (for example in allopatric populations) could result in reproductive divergence and eventually lead to speciation [44]–[46].

The identification of genes potentially involved in spermatogenesis and/or sperm-egg interactions will constitute an additional important advancement for understanding the reproductive biology of the medfly. Among insects, the testis transcriptome has been studied in detail only in *D. melanogaster* [47]–[50], *B. mori* [51], and in *A. gambiae* [52]. In the medfly, information about gene expression in the testes is almost completely lacking, the only exception being the studies on the testis-specific *β2 tubulin* gene [53] and partial data on the spermatogenesis and fertilization processes [54]–[56].

Here we describe the transcriptomes of the testes and accessory glands from adult male medfly to advance our understanding of the male reproductive system and the transcriptional profiles and putative roles of secreted candidates that may play a role in the regulation of female reproductive processes.

## Materials and Methods

### Medfly samples

**Figure 1:**
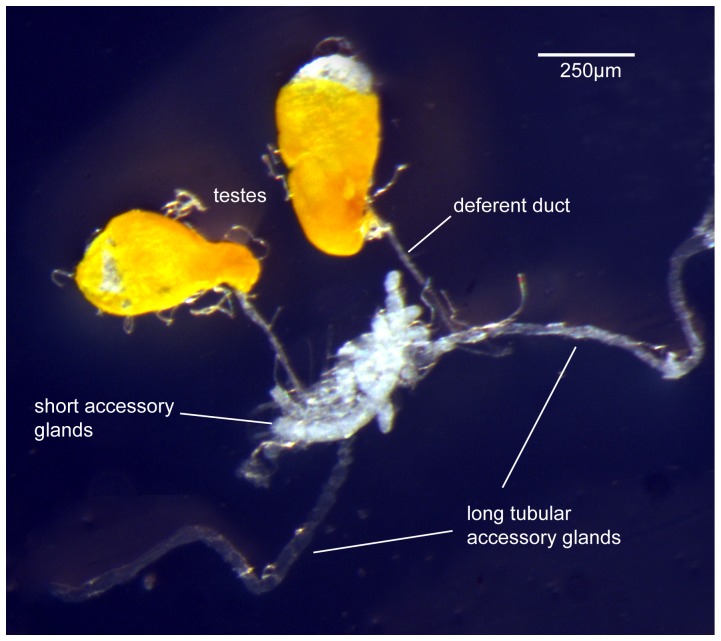
Dissection of the male reproductive tract including the testes and accessory glands.

An old established strain, ISPRA, which has been maintained at the University of Pavia since 1979, was used to create the cDNA library. Standard larval and rearing methods were used [57]. To obtain male adults for the testes and accessory glands (TAG) library, a standard laboratory rearing cage was set up with about 600 less than one day old male and female adults. Males were removed from the cage at intervals of 24 h for 8 days and the testes and MAGs were dissected in sterile PBS-DEPC (Figure 1). The dissected material was immediately immersed in *RNA-later* (Ambion) solution on ice and stored at −80°C until required. Thus the males used in the library construction covered a range of ages and consisted of immature (1–2 day old post emergence), virgin and mated individuals.

### cDNA library preparation and sequencing

For the cDNA library, total RNA was extracted from the testes and MAGs from each collection using Trizol (Invitrogen), followed by treatment with DNase (DNAfree, Ambion). An equal quantity of total RNA was pooled from each of the daily extractions prior to poly(A)^+^ RNA purification. First-strand cDNA synthesis was primed with an oligo(dT) containing a *Not*I restriction site. The double-stranded cDNA was ligated to an *EcoR*I adaptor, digested with *Not*I, and cloned directionally into a *Not*I- and *Eco*RI-digested pT7T3-Pac phagemid vector [58]. The cDNA inserts were flanked by a library-specific 3′ linker tag sequence (5′-*Not*I-TTGGCGGCGG-3′ and 5′ linker (5′-*EcoR*I-GGCACGAGG-3′). The library was normalized [58]. Randomly selected clones were sequenced from the 5′ end using the M13 reverse sequencing primer (5′-AGCGGATAACAATTTCACACAGGA-3′) with an Applied Biosystems 3730 DNA analyzer. Base-calling and low quality sequence trimming were performed using Phred [59], and vector sequences were trimmed using Cross-match (http://www.sanger.ac.uk/software/). Repeat sequences were masked using RepeatMasker (http://www.repeatmasker.org). The EST sequences have been deposited in GenBank dbEST database (accession numbers: JK832450–JK838363).

### Bioinformatics analyses

The sequences were assembled and annotated using the dCAS pipeline [60], [61]. The reads were clustered using BLAST [62], assembled into contigs using CAP3 [63] and annotated by searches against other databases. The results of these analyses were then piped into a hyperlinked Excel report, as described in the dCAS software tool [60].

The coding sequences (CDS) that were equal or larger than 40 amino acids (aa) were extracted according to two criteria i) matches to proteins in the NCBI nr database; ii) the largest ORF coincided with the same reading frame as a predicted signal peptide. Functional annotations of the transcripts were performed using the program Classifier (Ribeiro, unpublished) that combines the output of several tools: BLASTX [64] to compare the nucleotide sequences to the NCBI nr protein database, Swissprot, rpsblast [64] to search for conserved protein domains in Pfam [65], SMART [66], KOG [67], Conserved Domains Databases (CDD) [68] and GO databases [69]. Transcripts were also compared to mitochondrial and rRNA nucleotide sequences in NCBI. Transcripts that shared no significant sequence similarity using BLASTX were further analyzed using the more sensitive and rigorous FASTY program that uses the Smith-Waterman alignment algorithm [70] (http://fasta.bioch.virginia.edu/fasta_www2/fasta_list2.shtml). Segments of six-frame translations of transcripts with a methionine within the first 100 predicted amino acids were submitted to the SignalP server [71] to identify translation products that could be secreted. The SignalP analysis categorized the peptides as follows: SIG, predicted signal peptide by either the Neural Network or Hidden Markov Model programs; CYT, predicted cytoplasmic protein; BL, borderline result, where the probability of the predicted signal peptide was just below the acceptable threshold. In addition, TargetP predictions to nuclear and mitochondria protein destination were considered [72]. Since the signal peptide is often recognized as a membrane helix, TMHMM searches [73] were also performed, and translations with a predicted single helix within residue 30, which could correspond to the signal peptide membrane region, were also considered as target transcripts for further analyses. O-glycosylation sites on the proteins were predicted with the program NetOGlyc [74]. Gene ontology analysis was performed using Blast2GO (searches with an *e*<10^−6^) [75]. The tissue specificity of *Drosophila* orthologues of the identified transcripts was determined using the Flyatlas [76] expression database.

### Reverse Transcriptase-PCR on adult male individuals

The expression patterns of transcripts putatively encoding secreted proteins were determined by reverse transcription PCRs (RT-PCRs) using specific primers (Table S1) on cDNA derived from different body compartments. Four day-old virgin male and female medflies were separately dissected in PBS-DEPC onto glass slides and RNA was extracted from the following male tissues: 1) heads, 2) thoraces, 3) testes, 4) accessory glands (both long tubular and short claviform) and 5) abdomen without testes and MAGs, and the following female tissues: 6) heads, 7) thoraces and 8) abdomens. Extractions were performed using Trizol (Invitrogen), followed by treatment with DNase (Ambion), on pools of 50 individuals for each of the eight tissues. cDNA was synthesized from 200 ng RNA from each of the eight pools using the Cloned AMW First Strand Synthesis kit (Invitrogen) and one microlitre of the resulting first-stranded cDNA was then used as template for RT-PCR experiments. RT-PCRs were performed with the following thermal conditions: 2 min at 95°C, 25 cycles of 30 s at 95°C, 45 s at 59°C, 45 s at 72°C, and 10 min at 72°C. PCR products were analyzed on a 1% agarose gel.

### Real-Time quantitative PCR on virgin and mated males

Expression profiles were derived for candidate transcripts i) putatively encoding secreted proteins and ii) with specific expression, or significant over-expression, in male accessory glands. Expression levels of each transcript were tested in the abdomen of mature males, comparing virgin and mated individuals of same age to detect differences potentially induced by mating. The abdomen was used rather than the accessory glands and testes in these assays to avoid time-intensive dissections thus permitting the assessment of transcript abundance at the precise time intervals.

Virgin medfly individuals from the ISPRA strain were sexed after chilling at emergence and were used in mating assays on the fourth day after eclosion. All flies were maintained at 24°C with a 12∶12 h light/dark cycle on yeast-glucose medium before use in the experiments. For each of the three experimental replicates performed, 30 small cages (5×5×13 cm) were used, each containing one male and ten females, provided with food and water. Mating pair formation was observed from 08:00 to 20:00 h, thus covering the complete light period. Mating duration was recorded and only copulations longer than 100 min were considered to avoid false matings, i.e. those with little or no sperm transfer [77]. After a couple had separated, either the male was gently removed from the vial by aspiration or left inside and allowed to remate. Males were assigned to seven mating status groups according to the number of matings they had achieved and a recovery time after mating/remating of 0, 6 and 12 h, i.e.: 1 M+0 h, 1 M+6 h, 1 M+12 h, 2 M+0 h, 2 M+6 h, 2 M+12 h, 3 M+0 h (Figure S1). For example, the 2 M+6 h group consisted of males that had mated twice and had been allowed to recover for six hours after the termination of the last mating. For each mating status group, ten mated males were dissected and their abdomens immediately transferred to microcentrifuge tubes with 200 µl of Trizol and frozen at −80°C. As a control, a cage was set up containing about 100 virgin males of same age as those used for mating assays. Contemporary with each mated group, an equal number of unmated male abdomens from the control cage were collected in the same manner.

Extractions were performed using Trizol, followed by DNase treatment, on 10 pooled individuals for each of the seven mating status groups and for the corresponding seven unmated control groups. cDNA was synthesized from 200 ng RNA from each of the 14 pools, as described for the RT-PCR analyses. Primer pairs were designed to obtain amplification products of 100–250 bp, using *Beacon Designer 7* (Premier Biosoft International). The expression level of each transcript was determined for each of the seven time points between mated and virgin males of same age. The virgin male samples were taken as calibrators in order to assess the relative fold-change after mating. Two medfly housekeeping genes were used for relative quantification normalization: *CcActin* (GenBank acc. n. FG081771.1) and *CcRpL13A* (GenBank acc. n. FG085984.1) [78], [79]. Real-Time qPCR was performed using SsoFast™ EvaGreen® Supermix (Biorad). Cycling parameters were: 3 min at 95°C, 40 cycles of 10 sec at 95°C, 30 sec at 57°C and 30 sec at 68°C, and 10 min at 72°C. A fluorescence reading was made at the end of each extension step. Three replicates were performed and the specificity of the amplification product was determined by melt-curve analysis. PCR efficiencies were above 95% for all primer pairs. Relative quantification was performed using MJ Opticon Monitor™ Analysis Software 3.1.32 (Biorad). Data were analyzed using the Student's t-test. Results were expressed as mean ± standard error and a *P* value of <0.05 was considered statistically significant. In addition, a multivariate linear regression was applied to evaluate the time-dependency of gene transcription and to assess the impact of individual genes. We considered as dependent variable the log_2_ of the ratio of transcript abundance in mated and virgin samples. The independent predictors were the different time-points and the genes. As the data consisted of the means of replicated experiments, we exploited a weighted least square fit procedure [80], [81], where the vector of weights have been taken as the log_2_ of the ratio between the transcript abundance of mated samples and the corresponding standard error.

## Results

### Generation, assembly and functional annotation of the medfly testes and accessory gland ESTs

A unidirectional, normalized cDNA library was constructed from the testes and accessory glands (TAG) of immature, virgin and mated males ranging from less than one to eight days of age.

A total of 8448 cDNA clones were sequenced directionally from the 5′ end. These reads, once trimmed of vector, contaminants and low quality sequences, resulted in a total of 5914 high-quality sequence reads of average length 621 bp, amounting to almost four megabases of sequence. The ESTs were assembled into 3344 contiguous sequences (contigs, each with the prefix TAG), the highest number of ESTs in a single contig being 75. Nine hundred and fifty one contigs contained at least two ESTs, while 2393 contigs were represented by a single read (singleton). The distribution of the ESTs in the contigs is illustrated in Table 1.

**Table 1:** Medfly male testes and accessory gland EST assembly statistics.

Number of sequences	8448
Number of high quality masked sequences	5914
Number of assembled contigs	3344
Number of contigs containing:	
1 EST	2393
2–4 ESTs	727
5–10 ESTs	171
11–20 ESTs	41
21–30 ESTs	5
31–40 ESTs	3
>40 ESTs	4
Mean assembled sequence length (bp)	728
Mean contig length (bp)	830

**Figure 2:**
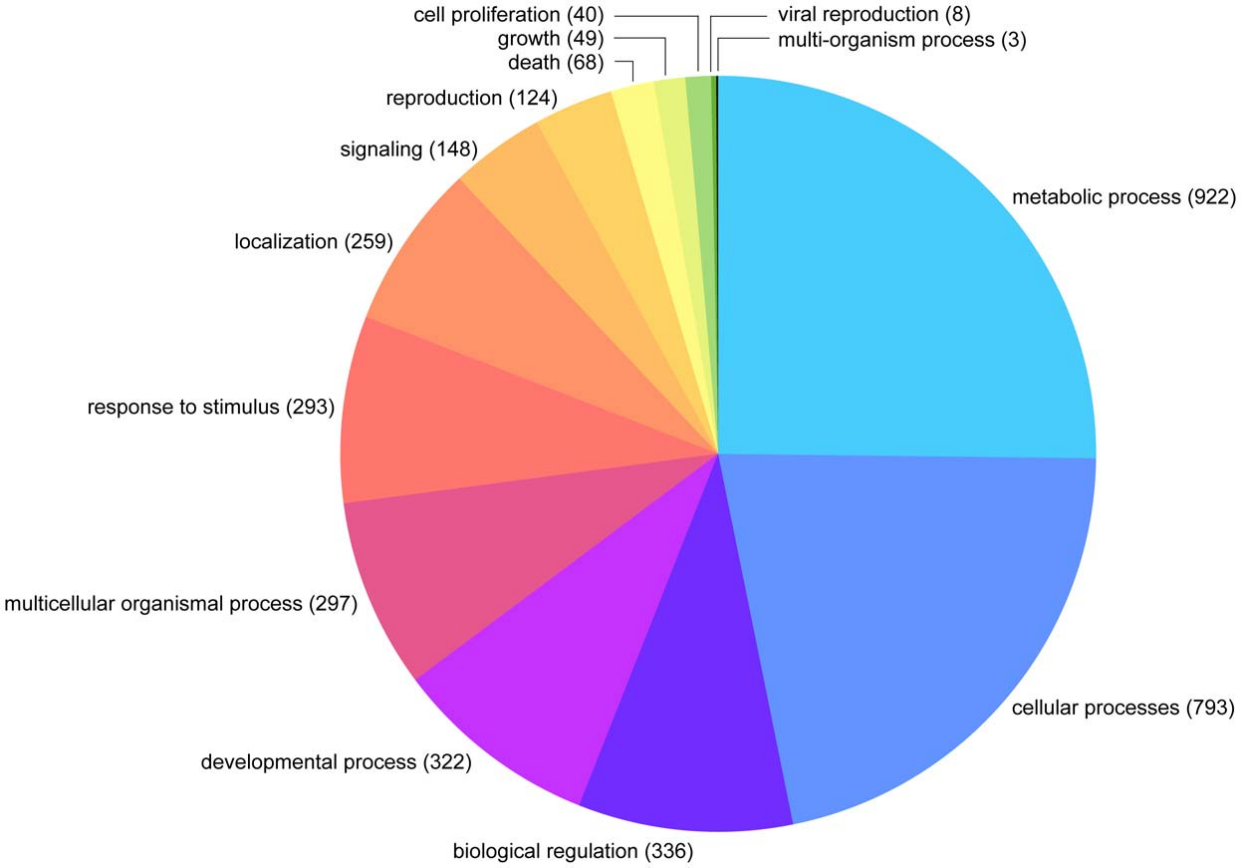
Distribution of the medfly TAG assembled sequences in Gene Ontology Biological Process categories Level II.

**Figure 3:**
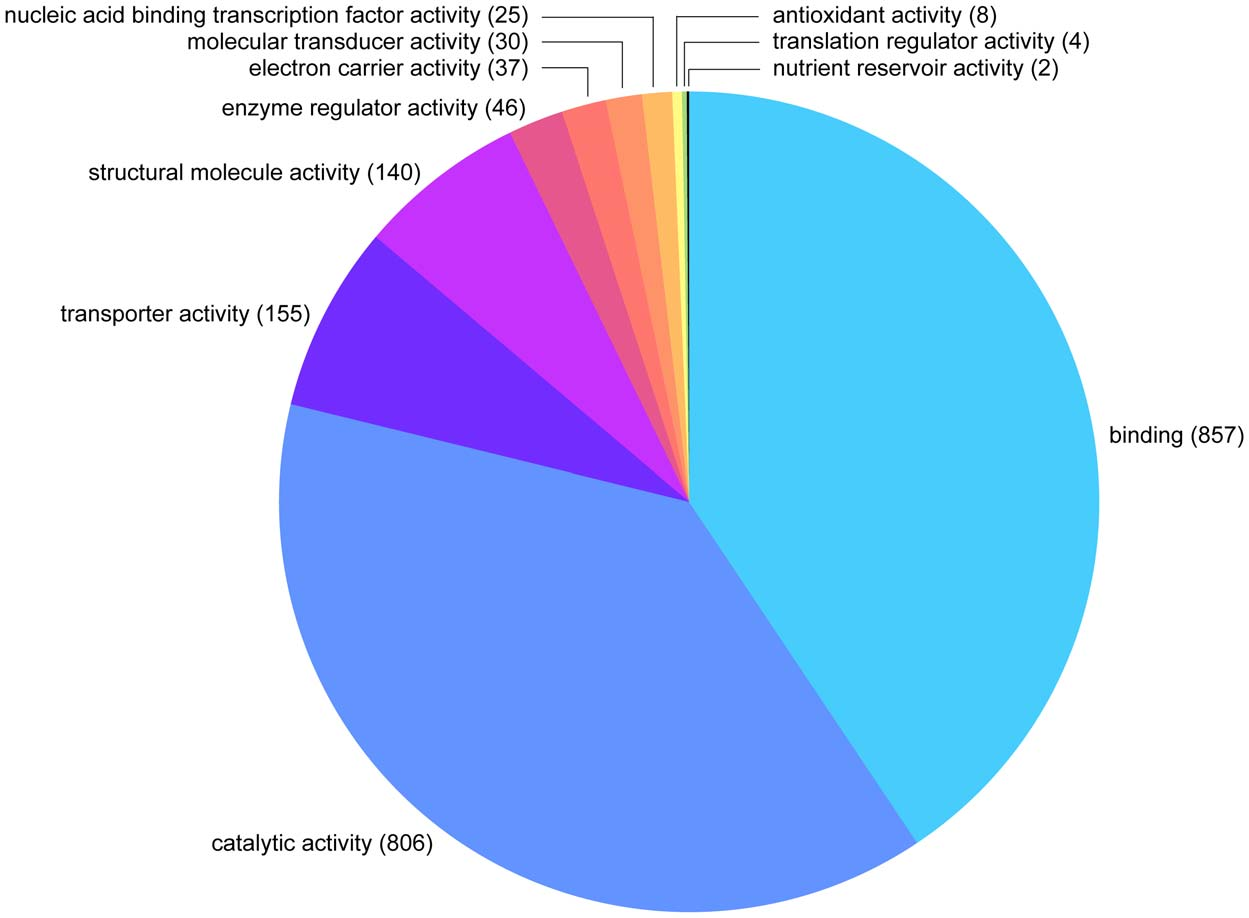
Distribution of the medfly TAG assembled sequences in Gene Ontology Molecular Function categories Level II.

Over 66% (2223) of the total number of assembled TAG sequences (3344) produced best hits against the nr database with an expectation (*e*) of <10^−6^ using BLASTX within Blast2GO. The vast majority (97%) of the best hits were arthropod-derived sequences, with *Drosophila* being the most common (75% of all hits), and only 1% (38) of the best hits were against known *C. capitata* sequences. Ten of the 13 putative MAG-specific transcripts previously identified in the medfly [29] were detected in the library. The remaining 33.5% (1121) of the assembled sequences produced no hits against the nr database and may putatively represent novel or rapidly evolving TAG sequences. Of the 2223 assembled sequences that gave significant hits, 1588 (71.4%) were categorized into different gene ontology (GO) classes according to biological process and molecular function (Figure 2, Figure 3).

Within the Biological Processes ontology, metabolic, cellular, and developmental process, and cellular component organization were the most representative terms. In addition, reproduction, biological regulation and response to stimulus terms were also abundant. In the Molecular Function ontology, the most abundant terms were binding and catalytic activities. Among the catalytic activity category, the hydrolase and transferase functions were the most frequent (Figure S2, Figure S3).

### Identification of the most abundant transcripts in the TAG transcriptome

As an initial description of the TAG transcriptome, we considered the 100 most abundant transcripts derived from the highest number of EST reads. This set included contigs that are derived from eight up to 75 reads. Ribosomal genes accounted for 35 of the 100 abundant sequences and the remaining 65 transcripts are reported in Table S2. Eight transcripts shared significant similarity to the medfly odorant-binding-protein-like, *male specific serum polypeptides* (MSSPs), and two (TAG1563 and TAG1565) to the *Drosophila odorant-binding protein 56d*. Moreover, the transcripts TAG1863 and TAG846 were similar to cytochrome c oxidase genes from the medfly and *Drosophila* respectively, while TAG640, TAG875 and TAG1003 shared similarity to the *Drosophila* cytoskeletal gene *alpha-tubulin 84B*, the fertility-related gene *exuperantia*, and a protease inhibitor gene of the grey fleshfly *Sarcophaga bullata*, respectively. By contrast, fifteen of the most abundant transcripts shared no sequence similarity to genes present in the GenBank database.

### Identification of putative peptides with secretory activity in the TAG transcriptome

Extraction of the coding sequences of the TAG transcripts and their conceptual translation products resulted in a total of 3371 putative peptides with a mean length of 167 residues. The number of peptide sequences is greater than the number of assembled sequences (3344), as several contigs yielded two very similar peptides that differed only in length. This redundancy in the dataset was maintained, as it was difficult to identify the correct peptide sequence when multiple methionine residues were present as start codons (Spreadsheet S1). The dCAS pipeline permitted the functional classification of the predicted peptides as shown in Table 2. In particular, we identified 2160 peptides with putative housekeeping functions (Table S3) and 400 with putative secretory activity (12% of total number of predicted peptides). These 400 peptides correspond to 304 TAG transcripts and include known gene families, such as odorant binding proteins (obps), protease inhibitor domain-containing peptides, antigen 5 proteins, mucins, and immunity-related sequences. However, 67% of the peptides classified as being associated with secretory function displayed no significant similarity to known proteins (Table 3). After excluding transcripts that were clearly not directly involved in reproduction, such as ribosomal and mitochondrial sequences, 206 transcripts that encoded putative secreted proteins (Spreadsheet S2) remained, and these were considered for expression profile analyses related to their tissue-specificity and mating-responsiveness. Among these were seven of the putative MAG-specific transcripts previously identified in the medfly [29].

**Table 2:** Functional classification and relative abundance of the TAG peptides.

Class	Number of contigs	%
Housekeeping	2160	64.08
Unknown	770	22.84
Putative secreted	400	11.87
Transposable elements	23	0.68
Bacterial	13	0.39
Viral	5	0.15
Total	3371	100

**Table 3:** Classification of TAG peptides with putative secretory function.

Putative secreted	Number of contigs	%
Odorant Binding Proteins	40	10
Protease inhibitor domains	20	5
Antigen 5 proteins	14	3.5
Mucins	12	3
Immunity related	10	2.5
Proteases	22	5.5
Other enzymes	12	3
Other putative secreted	270	67.5
Total	400	100

### Tissue-specific expression of TAG transcripts encoding putative secreted peptides

**Figure 4:**
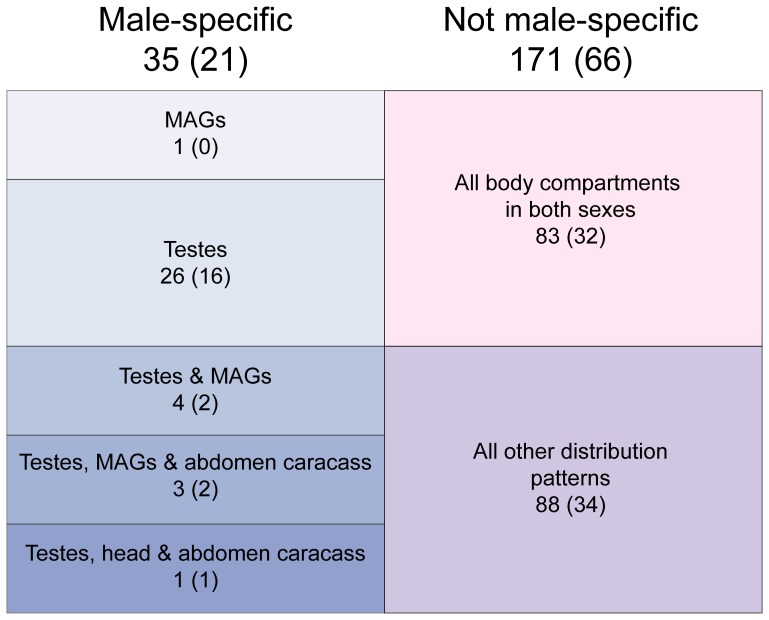
Tissue distribution of 206 transcripts that putatively encode secreted peptides as determined by RT-PCR on male and female body compartments. Figures in brackets indicate the number of transcripts that shared no significant similarity to known proteins.

The 206 candidate transcripts with putative secretory signals were assayed for tissue-specific expression patterns via RT-PCR using total RNA from different adult body compartments from males (heads, thoraces, testes, accessory glands and abdomen carcass (i.e. without testes and MAGs)), and from females (heads, thoraces and abdomens). Eighty-three transcripts were detected in both sexes in all the tissues examined, 88 were detected in different tissue patterns in both sexes, whilst 35 were found to have male-specific transcriptional profile (Figure 4, Table S4). Of these transcripts, 26 were specific to the testes and one to the MAGs (TAG40) (Table 4, Figure 4). The remaining eight male-specific transcripts displayed varying tissue distributions, with four expressed in the testes and MAGs (TAG1019, TAG1693, TAG2960, TAG3266), three in the testes, MAGs and abdomen carcass (TAG1692, TAG1695, TAG3261) and one in the testes, head and abdomen carcass (TAG2907). Interestingly, two additional transcripts, TAG1523 and TAG3324, despite being detected also in female tissues, displayed high transcriptional levels in the MAGs.

**Table 4:** Tissue distribution of 35 male-specific TAG transcripts encoding putative secreted peptides.

Tissue specificity	Transcript	Length (aa)	Best BLASTP hit against *Flybase*/NCBI nr^a^	e-value	Classification (GO Biological process)
Testes					
	TAG207	273	Antigen 5-related 2	3.1e-78	SCP-like domain
	TAG289	245	CG14840	4.0e-45	Extracellular matrix glyco-protein Laminin subunit beta (neurogenesis)
	TAG315	153	-	-	Hyp. secreted protein precursor
	TAG359	105	-	-	Hyp. secreted protein precursor
	TAG372	68	-	-	Hyp. secreted protein precursor
	TAG404	188	-	-	Hyp. secreted protein precursor
	TAG489	157	-	-	Hyp. secreted protein precursor
	TAG602	204	-	-	Hyp. secreted protein precursor
	TAG623	97	-	-	Hyp. secreted protein precursor
	TAG839	61	-	-	Hyp. secreted protein precursor
	TAG857	219	Sperm Leucylaminopeptidase 4	5.9e-104	Predicted aminopeptidase of the M17 family (proteolysis)
	TAG1111	148	B6DE45 *Anopheles darlingi* ^a^	8.0e-42	Hyp. secreted protein precursor
	TAG1117	184	CG12377	1.9e-37	Hyp. secreted protein precursor
	TAG1555	105	GA28049 *Drosophila p. pseudoobscura*	1.7e-13	Hyp. secreted protein precursor
	TAG1560	99	-	-	Hyp. secreted protein precursor
	TAG1570	90	-	-	Hyp. secreted protein precursor
	TAG1602	219	CG11286	6E-11	Hyp. secreted protein precursor
	TAG1902	48	-	-	Hyp. secreted protein precursor
	TAG2301	45	-	-	Hyp. secreted protein precursor
	TAG2321	162	-	-	Hyp. secreted protein precursor
	TAG2356	87	-	-	Hyp. secreted protein precursor
	TAG2860	155	CG13043	1.2e-22	Possible mucin
	TAG2889	57	-	-	Hyp. secreted protein precursor
	TAG3024	222	CG5217	7.5e-37	Hyp. secreted protein precursor
	TAG3102	218	CG12680	8.8e-49	Hyp. secreted protein precursor
	TAG3137	154	-	-	Hyp. secreted protein precursor
Testes, male head and male abdomen carcass					
	TAG2907	62	-	-	Hyp. secreted protein precursor
MAGs					
	TAG40^b^	334	CG5162	4.0e-119	Pancreatic lipase-like (lipid metabolic process)
MAGs and Testes					
	TAG1019	52	CG10853	3.5e-7	Hyp. secreted protein precursor
	TAG1693	156	-	-	Possible mucin
	TAG2960^c^	252	CG32271	7.6e-42	Trypsin-like serine protease (proteolysis)
	TAG3266^d^	95	-	-	Hyp. secreted protein precursor
MAGs, Testes and male abdomen carcass					
	TAG1692	154	-	-	possible mucin
	TAG1695	152	-	-	possible mucin
	TAG3261^e^	262	CG10407	3.4e-62	Haemolymph juvenile hormone binding protein

Four of these male-specific transcripts correspond to those previously identified [29],

b DQ406806,

c DQ406805,

d DQ406813,

e DQ406809.

The proportion of male-specific transcripts (21 of 35) that lacked significant similarity to known protein sequences was significantly greater than the proportion without hits in the non male specific transcripts (66 of 171) (Fisher's exact test, two tailed *P* = 0.0242).

The fourteen putative proteins encoded by the 35 male-specific transcripts that shared significant similarities to known protein sequences include ten testis-specific transcripts: TAG207 (similar to the *Drosophila antigen 5-related 2* and with a sperm coating protein-like domain), TAG289 (similar to CG14840), TAG857 (similar to the *sperm leucylaminopeptidase 4* and a predicted M17 family aminopeptidase), TAG1111 (similar to *Anopheles darlingi* B6DE45), TAG1117 (similar to CG12377), TAG1555 (similar to GA28049), TAG1602 (similar to CG11286), TAG2860 (similar to CG13043), TAG3024 (similar to CG5217) and TAG3102 (similar to CG12680) (Table 4). The MAG-specific transcript, TAG40, classified as a pancreatic lipase-like protein, is similar to CG5162. The two MAG and testes-enriched transcripts TAG1019 and TAG2960 are similar to CG10853 and a trypsin-like serine protease, respectively. One transcript detected in the MAGs, testes and abdomen carcass, TAG3261, shared similarity to a haemolymph juvenile hormone binding protein. Three other transcripts, TAG1692, TAG1693, and TAG1695, whilst lacking significant sequence similarity with known proteins, were classified as possible mucins based on the presence of potential glycosylation sites (also shown in Spreadsheet S2).

Four of the seven sequences that corresponded to the putative MAG-specific transcripts previously identified [29] were found to be male-specific, of which one (TAG40/DQ406806) was MAG-specific.

### Mating-induced transcriptional changes associated with genes encoding putative secreted proteins

Transcripts from ten genes with MAG-specific or -enriched expression profiles were analysed by Real-Time quantitative PCR to assess changes in their abundance after one, two or three matings, respectively. These included eight male-specific transcripts and two transcripts (TAG1523, TAG3324) with high abundance in the MAGs, but also present in other male and female tissues. The expression data were normalized relative to control virgin males of the same age similarly analysed at the 0, 6 and 12 h time points. Transcript TAG1019 showed very low abundance in all the treatments tested (CT>35) and was not further considered.

**Figure 5:**
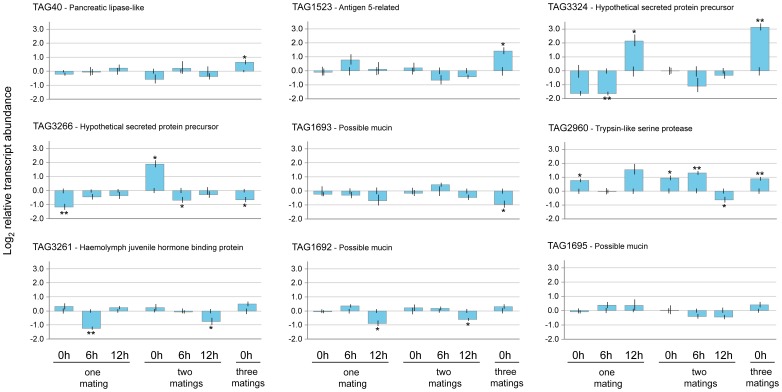
Differential transcript levels (Log_2_ transformed fold changes) of nine putative secreted peptides in mated medfly males, compared to virgin individuals of same age. Transcript abundances were determined at seven different time-points (0, 6 and 12 h after the first and the second mating respectively, and 0 h after the third copula) compared to virgin males of the same age. Stars indicate significant differences in transcript abundances (*P<0.05, **P<0.01, ***P<0.001, two-tailed *t*-test on three replicates) in the pairwise comparison between mated and virgin males.

The analysed genes showed very different and complex transcriptional profiles in response to one or more matings, suggesting potentially different functional roles (Figure 5). The two MAG-preferential transcripts, the putative pancreatic lipase TAG40 and the *antigen 5-related* TAG1523, showed a significant increase in abundance only after the third mating. Interestingly, the hypothetical secreted protein precursor TAG3324 displayed a significant decrease in abundance 6 h after the first mating, but its transcriptional activity subsequently increased. No significant differences relative to the virgins were detected for all the three time points following the second mating. The third mating, on the contrary, induced strong transcriptional activity. A similar decrease in transcript abundance after the first mating was detected for the hypothetical secreted protein, TAG3266, but its expression increased after the second mating, immediately *post copula*. However, its transcript levels significantly decreased 6 h after the second mating, as well as after the third mating. The first and second matings did not impact the transcriptional activity of the possible mucin TAG1693, while the third *copula* resulted in a reduction of transcript abundance. Concerning the putative trypsin-like serine protease TAG2960, transcript abundance was significantly higher in mated males relative to virgins immediately after each mating, with a decrease observed only 12 h after the second mating. The putative haemolymph juvenile hormone binding protein (TAG3261) showed significant reductions in transcript abundance 6 h after the first mating and 12 h after the second. Similarly, the possible mucin TAG1692 displayed decreases in abundance 12 h after both the first and the second copulation. Finally, the other possible mucin TAG1695 showed no significant changes in transcript abundance between virgin and mated males at all the time points analysed.

To evaluate the influence of the number of matings and recovery time on the transcriptional dynamics of the different genes, we applied a multivariate linear regression. This analysis revealed a statistically significant increase in transcriptional activity only immediately after the third mating (regression coefficient = 0.84, standard error = 0.35, *P* = 0.02). Furthermore, the gene TAG2960 had a statistically significant impact on the global gene transcript levels (regression coefficient = 0.92, SE = 0.41, *P* = 0.03).

## Discussion

Here we describe the first large-scale transcriptome dataset of 3344 sequences from the testes and male accessory glands of the medfly. What is more, it also represents the first transcript resource from these reproductive tissues in any tephritid species. This resource will be of vital importance not only for unravelling the reproductive processes of the medfly, but also for comparative evolutionary analyses of the reproductive molecular machinery between tephritid species and other more distant insect taxa. Within this transcriptome dataset, we have identified a subset of transcripts that, on the basis of their tissue-specificity, may encode medfly seminal fluid proteins. We described the transcriptional profiles of nine of these genes that showed mating-induced changes in abundance, most probably related to replenishment of their protein products after multiple matings.

### TAG transcriptome: conserved, novel and fast-evolving genes

The transcriptome dataset is derived from two organs, the testes and the accessory glands, that participate in the maintenance of complementary reproductive functions of the medfly male. On the basis of what is known from other insect taxa, we may suppose that, in the medfly testes, key regulatory genes of spermatogenesis tend to be conserved to guarantee the male-specific processes required for sperm production [82], [83]. The accessory gland secretions may act as key factors in male reproductive success and, as such, the Acp-encoding genes are subject to rapid evolution as a result of sexual conflict and competition [84]. Studies on numerous organisms from very distant taxa have shown that among the most rapidly evolving genes are those expressed in the reproductive tissues, particularly in males [85]–[91]. In the medfly, out of the 3344 unique transcripts we identified in the TAG library over a third shared no significant similarities to known genes and may be novel and/or fast-evolving sequences. They may have a potential role in sexual selection and speciation, and represent ideal subjects for future evolutionary genetic studies for this species. However, as this is the first extensive resource from the male reproductive tract in any tephritid species, many may be tephritid-specific rather than medfly-specific genes.

### Medfly testes and accessory glands express highly conserved genes that are critical for male reproduction across several insect species

Both testes and male accessory glands are sites of rapid cell proliferation and secretory activity, as supported by the categorization of transcripts in functional classes related to biological regulation, metabolic, developmental and cellular processes.

Several chemosensory-related transcripts are present in the TAG transcriptome. Ten putative peptides shared highly significant similarity to *D. melanogaster Obp99c* and also to the male specific serum polypeptides (MSSPs) previously characterized in the medfly [92], [93]. The MSSPs belong to the Minus-C subfamily of odorant binding proteins and are thought to be involved in the transport of volatile substances or other hydrophobic molecules [92], [93]. Two other transcripts (TAG1563 and TAG1565) showed high similarity to *Drosophila Obp56d*. Recent studies have reported the expression of obps in the male accessory glands and testes of *Drosophila* [76], [88], [94]–[96] and in the MAGs of the mosquito *A. aegypti* [6] and of *T. castaneum* [13]. The presence of obps in these tissues is not unexpected, as in addition to their function in olfaction, these proteins may also act as carriers for physiologically active ligands, such as hormones, that are transferred from the male to the female during copulation [96], [97].

Other highly abundant transcripts fall into categories involved in protein synthesis. The high representation of ribosomal transcripts is further indicative of the intense metabolic activity related to spermatogenesis, sperm membrane activity, and energy production and utilization. These functional categories are all consistent with the features of the sperm cell, which is rich in mitochondria and has a microtubule-based axoneme. Examples are the highly abundant *cytochrome c oxidase* transcripts, which may be involved in the energy production necessary for sperm locomotion and the *myosin light chain 2* orthologue, TAG698, which may cooperate in muscular activity. TAG875 is very similar to the *Drosophila* gene *exuperantia*, that encodes a germ-line restricted RNA-binding protein essential for fertility [98] and has been identified as one of the targets of *transformer-2* [99]. Another highly abundant transcript, TAG1003, shares similarity with a protease inhibitor and contains a Kunitz domain. The enzymatic reaction between a protease and its inhibitor is characterized by the formation of a pseudo-irreversible inhibitor-protease complex [100]. The protease inhibitor activity of some *Drosophila* Acps [101] and the homology of others to proteases [2], [10] suggest that some Acps play a role in the processing and activation of other Acps [102]–[104]. *Drosophila* seminal fluid also contains regulators of proteolysis, one being the trypsin inhibitor Acp62F that has been shown to enter the female's sperm storage organs to play a key role in protecting sperm from degradation [105]. During storage in the female organs, proteolysis of sperm surface proteins could impair the sperm's egg-binding ability. Alternatively, regulated proteolysis of the surface of stored sperm could be essential to activate or capacitate them. Indeed, in mice, mutations in seminal fluid protease inhibitors impair fertility, consistent with the hypothesis that protease inhibitors protect sperm [105]. Serpins (SERine Protease Inhibitors) have been shown to have a role in male fertility in many mammalian species [106]. In addition, data from *Caenorhabditis elegans* demonstrated the essential role of predicted secreted serine protease inhibitors for the regulation of sperm activation [107], [108]. This widespread presence of protease inhibitors across distant species supports the hypothesis of an important role of such molecules also in the medfly.

The best hit of TAG3302, *glutathione S-transferase*, is a predicted intracellular or membrane-bound protein [18]. Predicted intracellular proteins also have been reported in the seminal fluid of other organisms, such as *D. melanogaster* [109], bed bugs [110], honey bees [15], and humans [111]. In some species, including *A. mellifera* and *A. aegypti*, it has been suggested that these proteins may be secreted through non-standard secretion routes [15], [18], such as apocrine or holocrine secretion [112], [113]. For example, within the reproductive tract of mated *A. aegypti* females, the ejaculate contains vacuoles which grow after mating, and disappear within 24 h *post copula* [114]. Subunits of the membrane-bound proton ATPase thought to be part of these vacuoles have been identified as Acps [18]. Moreover, macroapocrine secretion has been reported in the medfly and the olive fly *Bactrocera oleae* [38], [115].

Elongation factors have also been identified in insect seminal fluids [110], [116]. For example, *elongation factor 1alpha* has been shown to be involved in protein synthesis, regulation of apoptosis and interaction with actin and ubiquitin-dependent proteolysis.

### The putative secreted TAG peptides belong to a series of functional classes characteristic of known seminal fluid proteins

Our expression analyses in different male and female adult tissues revealed that a third of the putative secreted peptides with testis-specific and/or MAG-enriched expression profiles belong to highly conserved Acp classes [117]. The medfly TAG putative secreted peptides belong to the CAP superfamily (Cysteine-Rich Secretory Proteins/Antigen 5/Pathogenesis-Related 1 Proteins), or are predicted to be mucins, proteases or lipases.

The remainder of our putative secreted peptides lack identifiable orthologues and this may reflect the pattern of rapid evolution described for a significant portion of Acps [7].

### *CAP (CRISP/Antigen5/PR-1) superfamily*

Proteins of the CAP family, also known as the sperm-coating glycoprotein (SCP) family, are secreted and several are known to be involved in male fertility and fertilization [118]. A number have been implicated in sperm chemo-attraction and sperm-egg fusion [119]. In *Drosophila*, the interaction of at least four SFPs, including the CRISP CG17575, is required for the binding of Acp70A to the sperm and to ensure their localisation in the female seminal receptacle [120]. Two medfly transcripts (TAG207 and TAG1523) are predicted members of the CAP superfamily, as they contain SCP domains and share sequence similarity to Ag5-related proteins [121]. TAG207 transcription was limited to the testes, whereas TAG1523 was abundant in the MAGs but also present in the heads of both sexes. Ag5 proteins were first identified within the venom of fire ants and wasps [122], and subsequently in the *Drosophila* midgut [123] and the saliva of bloodfeeding insects [124]–[126].

### *Mucins*

Four medfly putative TAG peptides shared similarities with proteins of the Mucin family, a group of large glycosylated macromolecules capable of forming enormous networks that act as selective barriers [127]. In *Drosophila*, mucins are expressed not only in the digestive tract, but also in the salivary glands and in the developing embryo, where they may contribute to the shaping of non-chitin-producing organs by providing a luminal scaffold during their development [127]. Additionally, mucins have been shown to participate, together with other proteins and lipids, in the formation of mating plugs, often produced within the female reproductive tract during or shortly after mating [7]. Mating plugs, which are present in a wide variety of organisms from insects to mammals have been proposed to play three main functions: to prevent remating, either as a physical barrier or by releasing chemical cues that prevent female remating; to favour sperm storage or to prevent sperm loss from the female reproductive tract; or to act as a visible signal of female mating status [128]–[131]. The medfly does not produce a plug, but mucins may have a sperm protection function, or may have a role in the differentiation and renewal of the epithelium and modulation of cell adhesion, immune response, and cell signalling [132], [133].

### *Proteases*

Proteases are present in the seminal fluid of many insect species and have been implicated in various aspects of male reproduction [7]. Seminal proteases could cleave inactive molecules into their active form, as in *D. melanogaster* Acp26Aa [104], or they could participate in the digestion or breakdown of mating plugs [129], [134]. Their presence in the transcriptomes of the reproductive tracts of both sexes is thus expected. Transcript TAG2960, which corresponds to the previously identified medfly MAG sequence DQ406805 [29], appears to encode a trypsin-like serine protease. This suggests that it is involved in proteolysis and, once secreted, it could be active within the male or female reproductive tract. In addition, TAG857 is a predicted aminopeptidase of the M17 family. In *Drosophila*, this family of leucyl aminopeptidases comprises sperm leucyl aminopeptidases (Sperm-LAPs, S-LAP) [135] that are specifically expressed in the testis and encode proteins incorporated in mature sperm [136]–[138].

### *Lipases*

High levels of lipase activity have previously been detected in male accessory glands of *Drosophila* [2], [10], *Phlebotomus papatasi* [139], and the medfly [29]. TAG40, which corresponds to the previously identified medfly MAG-specific sequence DQ406806 [29], shows similarity to lipases, that are postulated to provide energy to sperm by hydrolysis of triglycerides [109]. These lipases, once transferred to the female, may contribute nutritional resources, modify the chemistry of her reproductive tract to favour sperm and/or Acp function, or alter the sperm membrane to facilitate fertilization [2]. Carboxylesterase genes identified in the MAGs of *A. gambiae* and in *L. longipalpis* are homologues of *Drosophila* EST-6 [17], [19]. EST-6 in *Drosophila* is transferred to the female in the seminal fluid and influences oviposition behaviour and female receptivity to remating [140].

### Mating induces transcriptional changes in genes putatively encoding seminal fluid proteins

Five transcripts (TAG3266, TAG40, TAG1523, TAG2960 and TAG3324) showed significant changes in abundance after mating. Only TAG2960 appears to be a direct mating-responsive gene: it displays three distinct waves of transcriptional activity, immediately after each of the three matings. Due to its over-expression in the MAGs and its high similarity to a serine protease, it is an interesting medfly accessory gland protein candidate. In most of the other transcripts, the significant increase in abundance occurred after the third mating (examples are the MAG-specific TAG40 and TAG1523), suggesting that the mRNA (and perhaps the peptide) were not totally depleted after the first two copulations.

TAG1523, an *antigen 5-related* sequence, has previously been detected in the medfly MAGs [29]. It belongs to the large CAP family, involved in diverse functions, such as immune response [141] and testis and sperm development [142]. In *Drosophila*, several members of this family are preferentially expressed in males and some within primary spermatocytes [143]. It has been proposed that they may act either mediating interactions between germ-line and somatic cells within the male or between the sperm and egg [118]. The mating-responsiveness of *antigen 5-related* orthologue may be indicative of its involvement in gamete interactions also in the medfly.

TAG3324, together with TAG3266 (that corresponds to DQ406813 [29]), deserves further investigation due to the lack of detectable orthologues in other species. These two transcripts show significant changes in abundance at many time points. The oscillations in their abundance suggests switch points possibly related to the need to replenish transcripts encoding putative secreted proteins with an active role in the seminal fluid.

In several insects, juvenile hormone (JH) has been reported to accelerate the maturation of the male accessory glands [144]–[149] and is required for the renewal of secretory products depleted during mating [12], [144], [145], [147], [149], [150]. In *Drosophila*, JH not only contributes to the regulation of the initial accumulation and re-synthesis of accessory gland products [151]–[153], but it has also been proposed to promote male courtship [153]. Juvenile hormone is generally produced and released from the *corpora allata* [154], but in some species, such as *A. aegypti*, JH is synthesized *de novo* in the MAGs [155]. In spite of these roles of JH in insects, very little is known about its titre, function, and regulation in the medfly [156]. In another tephritid species, the Caribbean fruit fly, *Anastrepha suspensa*, mated males accumulate significantly higher levels of JHIII and JHB_3_ in the haemolymph than virgin males [157].

Juvenile hormone binding proteins (JHBPs) act as carriers of JH from the *corpora allata* to its target cells, and serve as a pool of JH in the haemolymph [158]. In addition, JHBPs protect JH from degradation by non-specific hydrolases [159]–[161]. The presence of JHBPs in the MAGs was previously reported in the medfly [29] and in *Drosophila* [162]. Acps synthesis can be stimulated by the ectopic application of JH [151], and putative JH binding sites have been identified upstream of the transcriptional start of Acp genes, suggesting a transcriptional regulation of some Acps by JH [151], [163], [164]. In the medfly, the transcript abundance of haemolymph JHBP (TAG3261, which corresponds to DQ406809 [29]) decreased significantly 6 h after the first mating and 12 h after the second mating, while at all other time points the abundance remained unchanged. In light of the complex regulation of JH in the insect reproductive biology, we hypothesize that these two significant drops in transcript abundance may be related to the replenishment of ejaculate components after mating. Studies on mating-induced changes in JH levels in male insects including the medfly are scarce, but in *Drosophila* it has been shown that JH levels increase after mating to stimulate Acps synthesis and to replenish the ejaculate components [165]. An increase in JH levels up-regulates the expression of juvenile hormone esterase (JHE), which, together with juvenile hormone epoxide hydrolase, hydrolyzes JH in order to regulate its levels [166], [167]. Once ejaculate replenishment is complete, JHE expression would reduce JH levels. On these bases, we hypothesize that the highly significant decrease of haemolymph JHBP (TAG3261) abundance several hours after mating may be related to this regulation of JH levels.

### The transcriptional activity of accessory gland genes is related to male mating behaviour

Some considerations emerge from the post-mating transcription profiles of genes that encode peptides in the male accessory glands in relation to the male mating behaviour. Our data indicate that for the majority of the considered genes there is no general increase in the transcriptional activity after each mating. However, after repeated copulations, and particularly after the third, their transcriptional profiles suggest that the depletion of their products presumably triggers transcription to replenish the proteins to be transferred. Thus, the availability of a reservoir of seminal fluid proteins sufficient for more than a single copulation is mirrored by the capacity of the male medfly to mate several times during the day [30]. The ability of the male to partition this reserve between successive females may be an efficient adaptive strategy to optimise his reproductive success. Moreover, the observation that the accessory gland proteins begin to be transferred within ten minutes after the start of copulation, long before the sperm, suggests that they may also be required to create the optimal physiological conditions in the female storage organs for the sperm [168]. As duration of copulation is not related to the quantity of sperm transferred to the female [77], the extended duration of this behaviour, which can last over five hours [77], is reminiscent of female guarding behaviour [169], [170]. This male strategy would prevent the female from remating with another male before the Acps had switched her behaviour towards oviposition.

In conclusion, the very complex transcriptional profiles of several of these genes suggest that they need to be further characterised. Clarification of seminal fluid components and their regulation will have the potential to reveal novel functions and processes associated with the reproductive biology of this pest species. In addition, studies of male recovery dynamics in terms of expression profiles of Acp genes, and the correlated mechanisms of female remating inhibition, may help improve pest management approaches.

## Supporting Information

Figure S1 **Schematic representation of the experimental design for Real-Time qPCR assays.** Once mating was completed, individual males (once mated) were removed and analysed at designated recovery times for gene expression (0, 6 and 12 h). The remaining males that had mated were immediately allowed to remate (twice mated) and treated as above. Remaining twice mated males were immediately allowed to mate again (thrice mated) and all were sacrificed after the completion of the *copula* (0 h). At each time point, virgin males of the same age as the mated males were likewise sacrificed as controls.
(TIF)

Figure S2 **Distribution of the medfly TAG assembled sequences in Gene Ontology Biological Process categories Level III.**
(TIF)

Figure S3 **Distribution of the medfly TAG assembled sequences in Gene Ontology Molecular Function categories Level III.**
(TIF)

Table S1 **Primers used in RT-PCR and Real-Time qPCR gene expression analyses.**
(DOC)

Table S2 **Descriptions of the 65 contigs derived from the most abundant transcripts in the medfly testes/male accessory glands transcriptome.**
(DOC)

Table S3 **Classification of TAG peptides with putative housekeeping functions.**
(DOC)

Table S4 **Tissue distribution of TAG transcripts that encode putative secreted peptides, as determined by RT-PCR.** Transcripts in bold gave significant hits against Flybase and/or the nr database (*e*<10−6).
(XLS)

Spreadsheet S1 **Hyperlinked Excel spreadsheet containing annotated assembled ESTs.** Putative peptides are named using the prefix ‘Cc-male-’ rather than the prefix ‘TAG’ used in the text.
(ZIP)

Spreadsheet S2 **Excel spreadsheet containing 206 putative secreted proteins considered for expression profile analyses related to their tissue-specificity and mating-responsiveness.**
(XLS)

## References

[1] Markow TA, Coppola A, Watts TD (2001) How *Drosophila* males make eggs: it is elemental. Proc Biol Sci 268: 1527–1532. 11454298 10.1098/rspb.2001.1673 PMC1088773

[2] Swanson WJ, Clark AG, Waldrip-Dail HM, Wolfner MF, Aquadro CF (2001) Evolutionary EST analysis identifies rapidly evolving male reproductive proteins in Drosophila. Proc Natl Acad Sci U S A 98: 7375–7379. 11404480 10.1073/pnas.131568198 PMC34676

[3] Gillott C (2003) Male accessory gland secretions: modulators of female reproductive physiology and behavior. Annu Rev Entomol 48: 163–184. 12208817 10.1146/annurev.ento.48.091801.112657

[4] Braswell WE, Andrés JA, Maroja LS, Harrison RG, Howard DJ, et al (2006) Identification and comparative analysis of accessory gland proteins in Orthoptera. Genome 49: 1069–1080. 17110987 10.1139/g06-061

[5] Poiani A (2006) Complexity of seminal fluid: a review. Behav Ecol Sociobiol 60: 289–310.

[6] Sirot LK, Poulson RL, McKenna MC, Girnary H, Wolfner MF, et al (2008) Identity and transfer of male reproductive gland proteins of the dengue vector mosquito, *Aedes aegypti*: potential tools for control of female feeding and reproduction. Insect Biochem Mol Biol 38: 176–189. 18207079 10.1016/j.ibmb.2007.10.007 PMC2758040

[7] Avila FW, Sirot LK, LaFlamme BA, Rubinstein CD, Wolfner MF (2011) Insect seminal fluid proteins: identification and function. Annu Rev Entomol 56: 21–40. 20868282 10.1146/annurev-ento-120709-144823 PMC3925971

[8] Wolfner MF, Applebaum S, Heifetz Y (2005) Insect gonadal glands and their gene products. In: Gilbert L, Iatrou K, Gill S, editors. Comprehensive Insect Physiology, Biochemistry, Pharmacology and Molecular Biology. Amsterdam: Elsevier. pp. 179–212.

[9] Wolfner MF (2007) “S.P.E.R.M.” (seminal proteins (are) essential reproductive modulators): the view from *Drosophila* . Soc Reprod Fertil Suppl 65: 183–199. 17644962

[10] Mueller JL, Ripoll DR, Aquadro CF, Wolfner MF (2004) Comparative structural modeling and inference of conserved protein classes in *Drosophila* seminal fluid. Proc Natl Acad Sci U S A 101: 13542–13547. 15345744 10.1073/pnas.0405579101 PMC518759

[11] Kelleher ES, Watts TD, LaFlamme BA, Haynes PA, Markow TA (2009) Proteomic analysis of *Drosophila mojavensis* male accessory glands suggests novel classes of seminal fluid proteins. Insect Biochem Mol Biol 39: 366–371. 19328853 10.1016/j.ibmb.2009.03.003

[12] Parthasarathy R, Tan A, Sun Z, Chen Z, Rankin M, et al (2009) Juvenile hormone regulation of male accessory gland activity in the red flour beetle, Tribolium castaneum. Mech Dev 126: 563–579. 19324087 10.1016/j.mod.2009.03.005 PMC2739235

[13] South A, Sirot LK, Lewis SM (2011) Identification of predicted seminal fluid proteins in *Tribolium castaneum* . Insect Mol Biol 20: 447–456. 21689183 10.1111/j.1365-2583.2011.01083.x

[14] Walters JR, Harrison RG (2010) Combined EST and proteomic analysis identifies rapidly evolving seminal fluid proteins in *Heliconius* butterflies. Mol Biol Evol 27: 2000–2013. 20375075 10.1093/molbev/msq092

[15] Baer B, Heazlewood JL, Taylor NL, Eubel H, Millar AH (2009) The seminal fluid proteome of the honeybee Apis mellifera. Proteomics 9: 2085–2097. 19322787 10.1002/pmic.200800708

[16] Oppelt A, Humann FC, Fuessl M, Azevedo SV, Marco Antonio DS, et al (2010) Suppression subtractive hybridization analysis reveals expression of conserved and novel genes in male accessory glands of the ant *Leptothorax gredleri* . BMC Evol Biol 10: 273. 20825642 10.1186/1471-2148-10-273 PMC2949867

[17] Azevedo RV, Dias DB, Bretãs JA, Mazzoni CJ, Souza NA, et al (2012) The transcriptome of Lutzomyia longipalpis (Diptera: Psychodidae) male reproductive organs. PLoS One 7: e34495. 22496818 10.1371/journal.pone.0034495 PMC3320635

[18] Sirot LK, Hardstone MC, Helinski ME, Ribeiro JM, Kimura M, et al (2011) Towards a semen proteome of the dengue vector mosquito: protein identification and potential functions. PLoS Negl Trop Dis 5: e989. 21423647 10.1371/journal.pntd.0000989 PMC3057948

[19] Dottorini T, Nicolaides L, Ranson H, Rogers DW, Crisanti A, et al (2007) A genome-wide analysis in Anopheles gambiae mosquitoes reveals 46 male accessory gland genes, possible modulators of female behavior. Proc Natl Acad Sci U S A 104: 16215–16220. 17901209 10.1073/pnas.0703904104 PMC2042187

[20] Kern AD, Jones CD, Begun DJ (2004) Molecular population genetics of male accessory gland proteins in the Drosophila simulans complex. Genetics 167: 725–735. 15238524 10.1534/genetics.103.020883 PMC1470896

[21] Harris EJ (1989) Pest status of fruit flies. In: Robinson AS, Hooper GH, editors. Fruit Flies: Their Biology, Natural Enemies and Control. Amsterdam: Elsevier. pp. 73–81.

[22] Malacrida AR, Gomulski LM, Bonizzoni M, Bertin S, Gasperi G, et al (2007) Globalization and fruitfly invasion and expansion: the medfly paradigm. Genetica 131: 1–9. 17111234 10.1007/s10709-006-9117-2

[23] Gomulski LM, Dimopoulos G, Xi Z, Soares MB, Bonaldo MF, et al (2008) Gene discovery in an invasive tephritid model pest species, the Mediterranean fruit fly, *Ceratitis capitata* . BMC Genomics 9: 243. 18500975 10.1186/1471-2164-9-243 PMC2427042

[24] Carey JR, Vaupel JW (2003) Biodemography. In: Delamater J, editor. Handbook of the Social Phycology. New York: Kluwer Academic/Plenum Publisher. pp. 625–658.

[25] Papadopoulos NT, Liedo P, Müller HG, Wang JL, Molleman F, et al (2010) Cost of reproduction in male medflies: the primacy of sexual courting in extreme longevity reduction. J Insect Physiol 56: 283–287. 19896949 10.1016/j.jinsphys.2009.10.014 PMC3018851

[26] Theodoraki M, Tatari M, Chrysanthis G, Zacharopoulou A, Mintzas AC (2008) Structural characterization of the medfly hsp83 gene and functional analysis of its proximal promoter region in vivo by germ-line transformation. Arch Insect Biochem Physiol 67: 20–35. 18064699 10.1002/arch.20216

[27] Gabrieli P, Falaguerra A, Siciliano P, Gomulski LM, Scolari F, et al (2010) Sex and the single embryo: early deveiopment in the Mediterranean fruit fly, Ceratitis capitata. BMC Dev Biol 10: 12. 20102629 10.1186/1471-213X-10-12 PMC2826288

[28] Gomulski LM, Dimopoulos G, Xi Z, Scolari F, Gabrieli P, et al (2012) Transcriptome profiling of sexual maturation and mating in the Mediterranean fruit fly, Ceratitis capitata. PLoS One 7: e30857. 22303464 10.1371/journal.pone.0030857 PMC3267753

[29] Davies SJ, Chapman T (2006) Identification of genes expressed in the accessory glands of male Mediterranean Fruit Flies (*Ceratitis capitata*). Insect Biochem Mol Biol 36: 846–856. 17046598 10.1016/j.ibmb.2006.08.009

[30] Cavalloro R, Delrio G (1970) Studi sulla radiosterilizzazione di *Ceratitis capitata* Wiedemann e sul comportamento dell'insetto normale e sterile. Redia LII: 511–547.

[31] Delrio G, Cavalloro R (1979) Influenza dell'accoppiamento sulla recettività sessuale e sull'ovideposizione in femmine di C*eratitis capitata* Wiedemann. Entomologica XV: 127–143.

[32] Miyatake T, Chapman T, Partridge L (1999) Mating-induced inhibition of remating in female Mediterranean fruit flies Ceratitis capitata. J Insect Physiol 45: 1021–1028. 12770277 10.1016/s0022-1910(99)00083-9

[33] Jang EB (1995) Effects of mating and accessory-gland injections on olfactory-mediated behavior in the female Mediterranean fruit-fly, *Ceratitis capitata* . J Ins Physiol 41: 705–710.

[34] Moshitzky P, Gilbert LI, Applebaum SW (2003) Biosynthetic maturation of the *corpus allatum* of the female adult medfly, *Ceratitis capitata*, and its putative control. J Insect Physiol 49: 603–609. 12804720 10.1016/s0022-1910(03)00047-7

[35] Chen PS, Stumm-Zollinger E, Aigaki T, Balmer J, Bienz M, et al (1988) A male accessory gland peptide that regulates reproductive behavior of female *D. melanogaster* . Cell 54: 291–298. 3135120 10.1016/0092-8674(88)90192-4

[36] Swanson WJ (2003) Sex peptide and the sperm effect in *Drosophila melanogaster* . Proc Natl Acad Sci U S A 100: 9643–9644. 12913117 10.1073/pnas.1834127100 PMC187794

[37] Liu H, Kubli E (2003) Sex-peptide is the molecular basis of the sperm effect in *Drosophila melanogaster* . Proc Natl Acad Sci U S A 100: 9929–9933. 12897240 10.1073/pnas.1631700100 PMC187889

[38] Marchini D, Del Bene G, Cappelli L, Dallai R (2003) Ultrastructure of the male reproductive accessory glands in the medfly Ceratitis capitata (Diptera: Tephritidae) and preliminary characterization of their secretions. Arthropod Struct Dev 31: 313–327. 18088989 10.1016/S1467-8039(03)00003-3

[39] Mueller JL, Ravi Ram K, McGraw LA, Bloch Qazi MC, Siggia ED, et al (2005) Cross-species comparison of *Drosophila* male accessory gland protein genes. Genetics 171: 131–143. 15944345 10.1534/genetics.105.043844 PMC1456506

[40] Begun DJ, Lindfors HA, Thompson ME, Holloway AK (2006) Recently evolved genes identified from *Drosophila yakuba* and *D. erecta* accessory gland expressed sequence tags. Genetics 172: 1675–1681. 16361246 10.1534/genetics.105.050336 PMC1456303

[41] Levine MT, Jones CD, Kern AD, Lindfors HA, Begun DJ (2006) Novel genes derived from noncoding DNA in *Drosophila melanogaster* are frequently X-linked and exhibit testis-biased expression. Proc Natl Acad Sci U S A 103: 9935–9939. 16777968 10.1073/pnas.0509809103 PMC1502557

[42] Schully SD, Hellberg ME (2006) Positive selection on nucleotide substitutions and indels in accessory gland proteins of the *Drosophila pseudoobscura* subgroup. J Mol Evol 62: 793–802. 16752217 10.1007/s00239-005-0239-4

[43] Andrés JA, Maroja LS, Harrison RG (2008) Searching for candidate speciation genes using a proteomic approach: seminal proteins in field crickets. Proc Biol Sci 275: 1975–1983. 18495616 10.1098/rspb.2008.0423 PMC2596363

[44] Andrés JA, Arnqvist G (2001) Genetic divergence of the seminal signal-receptor system in houseflies: the footprints of sexually antagonistic coevolution? Proc Biol Sci 268: 399–405. 11270437 10.1098/rspb.2000.1392 PMC1088620

[45] Coyne JA, Orr HA (2004) Speciation. Sunderland, MA: Sinauer Associates.

[46] Fricke C, Arnqvist G, Amaro N (2006) Female modulation of reproductive rate and its role in postmating prezygotic isolation in *Callosobruchus maculatus* . Funct Ecol 20: 360–368.

[47] Andrews J, Bouffard GG, Cheadle C, Lü J, Becker KG, et al (2000) Gene discovery using computational and microarray analysis of transcription in the *Drosophila melanogaster* testis. Genome Res 10: 2030–2043. 11116097 10.1101/gr.10.12.2030 PMC313064

[48] Parisi M, Nuttall R, Naiman D, Bouffard G, Malley J, et al (2003) Paucity of genes on the *Drosophila* X chromosome showing male-biased expression. Science 299: 697–700. 12511656 10.1126/science.1079190 PMC1363366

[49] Ranz JM, Castillo-Davis CI, Meiklejohn CD, Hartl DL (2003) Sex-dependent gene expression and evolution of the *Drosophila* transcriptome. Science 300: 1742–1745. 12805547 10.1126/science.1085881

[50] Mikhaylova LM, Nguyen K, Nurminsky DI (2008) Analysis of the *Drosophila melanogaster* testes transcriptome reveals coordinate regulation of paralogous genes. Genetics 179: 305–315. 18493055 10.1534/genetics.107.080267 PMC2390609

[51] Arunkumar KP, Mita K, Nagaraju J (2009) The silkworm Z chromosome is enriched in testis-specific genes. Genetics 182: 493–501. 19332883 10.1534/genetics.108.099994 PMC2691758

[52] Krzywinska E, Krzywinski J (2009) Analysis of expression in the *Anopheles gambiae* developing testes reveals rapidly evolving lineage-specific genes in mosquitoes. BMC Genomics 10: 300. 19580678 10.1186/1471-2164-10-300 PMC2713267

[53] Scolari F, Schetelig MF, Bertin S, Malacrida AR, Gasperi G, et al (2008) Fluorescent sperm marking to improve the fight against the pest insect *Ceratitis capitata* (Wiedemann; Diptera: Tephritidae). N Biotechnol 25: 76–84. 18504022 10.1016/j.nbt.2008.02.001

[54] Pasini ME, Intra J, Gomulski LM, Calvenzani V, Petroni K, et al (2011) Identification and expression profiling of *Ceratitis capitata* genes coding for β-hexosaminidases. Gene 473: 44–56. 21094225 10.1016/j.gene.2010.11.003

[55] Intra J, De Caro D, Perotti ME, Pasini ME (2011) Glycosidases in the plasma membrane of *Ceratitis capitata* spermatozoa. Insect Biochem Mol Biol 41: 90–100. 21044684 10.1016/j.ibmb.2010.10.004

[56] Báo SN, Quagio-Grassiotto I, Dolder H (1989) Acrosome formation in *Ceratitis capitata* (Diptera, Tephritidae). Cytobios 58: 93–100. 2805814

[57] Saul SH (1982) Rosy-like mutant of the Mediterranean fruit fly, *Ceratitis capitata* (Diptera: Tephritidae), and its potential for use in a genetic sexing program. Ann Entomol Soc Am 75: 480–483.

[58] Bonaldo MF, Lennon G, Soares MB (1996) Normalization and subtraction: two approaches to facilitate gene discovery. Genome Res 6: 791–806. 8889548 10.1101/gr.6.9.791

[59] Ewing B, Hillier L, Wendl MC, Green P (1998) Base-calling of automated sequencer traces using phred. I. Accuracy assessment. Genome Res 8: 175–185. 9521921 10.1101/gr.8.3.175

[60] Guo Y, Ribeiro JM, Anderson JM, Bour S (2009) dCAS: a desktop application for cDNA sequence annotation. Bioinformatics 25: 1195–1196. 19318425 10.1093/bioinformatics/btp129 PMC2732306

[61] Ribeiro JM, Labruna MB, Mans BJ, Maruyama SR, Francischetti IM, et al (2012) The sialotranscriptome of *Antricola delacruzi* female ticks is compatible with non-hematophagous behavior and an alternative source of food. Insect Biochem Mol Biol 42: 332–342. 22306723 10.1016/j.ibmb.2012.01.003 PMC3351099

[62] Altschul SF, Gish W, Miller W, Myers EW, Lipman DJ (1990) Basic local alignment search tool. J Mol Biol 215: 403–410. 2231712 10.1016/S0022-2836(05)80360-2

[63] Kumar S, Tamura K, Nei M (2004) MEGA3: Integrated software for Molecular Evolutionary Genetics Analysis and sequence alignment. Brief Bioinform 5: 150–163. 15260895 10.1093/bib/5.2.150

[64] Altschul SF, Madden TL, Schäffer AA, Zhang J, Zhang Z, et al (1997) Gapped BLAST and PSI-BLAST: a new generation of protein database search programs. Nucleic Acids Res 25: 3389–3402. 9254694 10.1093/nar/25.17.3389 PMC146917

[65] Bateman A, Birney E, Durbin R, Eddy SR, Howe KL, et al (2000) The Pfam protein families database. Nucleic Acids Res 28: 263–266. 10592242 10.1093/nar/28.1.263 PMC102420

[66] Schultz J, Milpetz F, Bork P, Ponting CP (1998) SMART, a simple modular architecture research tool: identification of signaling domains. Proc Natl Acad Sci U S A 95: 5857–5864. 9600884 10.1073/pnas.95.11.5857 PMC34487

[67] Tatusov RL, Fedorova ND, Jackson JD, Jacobs AR, Kiryutin B, et al (2003) The COG database: an updated version includes eukaryotes. BMC Bioinformatics 4: 41. 12969510 10.1186/1471-2105-4-41 PMC222959

[68] Marchler-Bauer A, Anderson JB, DeWeese-Scott C, Fedorova ND, Geer LY, et al (2003) CDD: a curated Entrez database of conserved domain alignments. Nucleic Acids Res 31: 383–387. 12520028 10.1093/nar/gkg087 PMC165534

[69] Ashburner M, Ball CA, Blake JA, Botstein D, Butler H, et al (2000) Gene ontology: tool for the unification of biology. The Gene Ontology Consortium. Nat Genet 25: 25–29. 10802651 10.1038/75556 PMC3037419

[70] Pearson WR, Wood T, Zhang Z, Miller W (1997) Comparison of DNA sequences with protein sequences. Genomics 46: 24–36. 9403055 10.1006/geno.1997.4995

[71] Nielsen H, Engelbrecht J, Brunak S, von Heijne G (1997) Identification of prokaryotic and eukaryotic signal peptides and prediction of their cleavage sites. Protein Eng 10: 1–6. 10.1093/protein/10.1.1 9051728

[72] Emanuelsson O, Nielsen H, Brunak S, von Heijne G (2000) Predicting subcellular localization of proteins based on their N-terminal amino acid sequence. J Mol Biol 300: 1005–1016. 10891285 10.1006/jmbi.2000.3903

[73] Möller S, Croning MD, Apweiler R (2001) Evaluation of methods for the prediction of membrane spanning regions. Bioinformatics 17: 646–653. 11448883 10.1093/bioinformatics/17.7.646

[74] Hansen JE, Lund O, Tolstrup N, Gooley AA, Williams KL, et al (1998) NetOglyc: prediction of mucin type O-glycosylation sites based on sequence context and surface accessibility. Glycoconj J 15: 115–130. 9557871 10.1023/a:1006960004440

[75] Conesa A, Gotz S, Garcia-Gomez J, Terol J, Talon M, et al (2005) Blast2GO: a universal tool for annotation, visualization and analysis in functional genomics research. Bioinformatics 21: 3674–3676. 16081474 10.1093/bioinformatics/bti610

[76] Chintapalli VR, Wang J, Dow JA (2007) Using FlyAtlas to identify better *Drosophila melanogaster* models of human disease. Nat Genet 39: 715–720. 17534367 10.1038/ng2049

[77] Taylor PW, Yuval B (1999) Postcopulatory sexual selection in Mediterranean fruit flies: advantages for large and protein-fed males. Anim Behav 58: 247–254. 10458875 10.1006/anbe.1999.1137

[78] Vandesompele J, De Preter K, Pattyn F, Poppe B, Van Roy N, et al (2002) Accurate normalization of real-time quantitative RT-PCR data by geometric averaging of multiple internal control genes. Genome Biol 3: RESEARCH0034. 12184808 10.1186/gb-2002-3-7-research0034 PMC126239

[79] Scharlaken B, de Graaf DC, Goossens K, Brunain M, Peelman LJ, et al (2008) Reference gene selection for insect expression studies using quantitative real-time PCR: The head of the honeybee, *Apis mellifera*, after a bacterial challenge. J Insect Sci 8: 33.

[80] Wolberg J (2005) Data Analysis Using the Method of LeastSquares: Extracting the Most Information from Experiments. Berlin Heidelberg New York Springer Verlag

[81] Team RDC (2011) R: A language and environment for statistical computing. Vienna, Austria:R Foundation for Statistical Computing.

[82] Bonilla E, Xu EY (2008) Identification and characterization of novel mammalian spermatogenic genes conserved from fly to human. Mol Hum Reprod 14: 137–142. 18256174 10.1093/molehr/gan002

[83] White-Cooper H, Doggett K, Ellis R (2009) The evolution of spermatogenesis. In: Birkhead TR, Hosken DJ, Pitnick SS, editors. Sperm biology: an evolutionary perspective. New York: Academic Press. pp. 151–183.

[84] Ravi Ram K, Wolfner MF (2007) Sustained post-mating response in *Drosophila melanogaster* requires multiple seminal fluid proteins. PLoS Genet 3: e238. 18085830 10.1371/journal.pgen.0030238 PMC2134937

[85] Wong A, Turchin M, Wolfner MF, Aquadro CF (2012) Temporally variable selection on proteolysis-related reproductive tract proteins in Drosophila. Mol Biol Evol 29: 229–238. 21940639 10.1093/molbev/msr197 PMC3283112

[86] Panhuis TM, Clark NL, Swanson WJ (2006) Rapid evolution of reproductive proteins in abalone and Drosophila. Philos Trans R Soc Lond B Biol Sci 361: 261–268. 16612885 10.1098/rstb.2005.1793 PMC1569613

[87] Clark NL, Aagaard JE, Swanson WJ (2006) Evolution of reproductive proteins from animals and plants. Reproduction 131: 11–22. 16388004 10.1530/rep.1.00357

[88] Chapman T (2008) The soup in my fly: evolution, form and function of seminal fluid proteins. PLoS Biol 6: e179. 18666830 10.1371/journal.pbio.0060179 PMC2486303

[89] Swanson WJ, Vacquier VD (2002) The rapid evolution of reproductive proteins. Nat Rev Genet 3: 137–144. 11836507 10.1038/nrg733

[90] Civetta A (2003) Positive selection within sperm-egg adhesion domains of fertilin: an ADAM gene with a potential role in fertilization. Mol Biol Evol 20: 21–29. 12519902 10.1093/molbev/msg002

[91] Haerty W, Jagadeeshan S, Kulathinal RJ, Wong A, Ravi Ram K, et al (2007) Evolution in the fast lane: rapidly evolving sex-related genes in Drosophila. Genetics 177: 1321–1335. 18039869 10.1534/genetics.107.078865 PMC2147986

[92] Christophides GK, Mintzas AC, Komitopoulou K (2000) Organization, evolution and expression of a multigene family encoding putative members of the odourant binding protein family in the medfly Ceratitis capitata. Insect Mol Biol 9: 185–195. 10762426 10.1046/j.1365-2583.2000.00176.x

[93] Christophides GK, Livadaras I, Savakis C, Komitopoulou K (2000) Two medfly promoters that have originated by recent gene duplication drive distinct sex, tissue and temporal expression patterns. Genetics 156: 173–182. 10978283 10.1093/genetics/156.1.173 PMC1461254

[94] Allen AK, Spradling AC (2008) The Sf1-related nuclear hormone receptor Hr39 regulates *Drosophila* female reproductive tract development and function. Development 135: 311–321. 18077584 10.1242/dev.015156

[95] Yamamoto MT, Takemori N (2010) Proteome profiling reveals tissue-specific protein expression in the male reproductive system of *Drosophila melanogaster* . Fly (Austin) 4: 36–39. 20139713 10.4161/fly.4.1.10838

[96] Zhou S, Stone EA, Mackay TF, Anholt RR (2009) Plasticity of the chemoreceptor repertoire in *Drosophila melanogaster* . PLoS Genet 5: e1000681. 19816562 10.1371/journal.pgen.1000681 PMC2750752

[97] Arya GH, Weber AL, Wang P, Magwire MM, Negron YL, et al (2010) Natural variation, functional pleiotropy and transcriptional contexts of odorant binding protein genes in Drosophila melanogaster. Genetics 186: 1475–1485. 20870963 10.1534/genetics.110.123166 PMC2998325

[98] Hazelrigg T, Watkins WS, Marcey D, Tu C, Karow M, et al (1990) The *exuperantia* gene is required for *Drosophila* spermatogenesis as well as anteroposterior polarity of the developing oocyte, and encodes overlapping sex-specific transcripts. Genetics 126: 607–617. 2249760 10.1093/genetics/126.3.607 PMC1204216

[99] Hazelrigg T, Tu C (1994) Sex-specific processing of the *Drosophila exuperantia* transcript is regulated in male germ cells by the *tra-2* gene. Proc Natl Acad Sci U S A 91: 10752–10756. 7938024 10.1073/pnas.91.22.10752 PMC45100

[100] Laskowski M, Kato I (1980) Protein inhibitors of proteinases. Annu Rev Biochem 49: 593–626. 6996568 10.1146/annurev.bi.49.070180.003113

[101] Lung O, Tram U, Finnerty CM, Eipper-Mains MA, Kalb JM, et al (2002) The *Drosophila melanogaster* seminal fluid protein Acp62F is a protease inhibitor that is toxic upon ectopic expression. Genetics 160: 211–224. 11805057 10.1093/genetics/160.1.211 PMC1461949

[102] Park M, Wolfner MF (1995) Male and female cooperate in the prohormone-like processing of a *Drosophila melanogaster* seminal fluid protein. Dev Biol 171: 694–702. 7556947 10.1006/dbio.1995.1315

[103] Monsma SA, Harada HA, Wolfner MF (1990) Synthesis of two *Drosophila* male accessory gland proteins and their fate after transfer to the female during mating. Dev Biol 142: 465–475. 2257979 10.1016/0012-1606(90)90368-s

[104] Heifetz Y, Vandenberg LN, Cohn HI, Wolfner MF (2005) Two cleavage products of the Drosophila accessory gland protein ovulin can independently induce ovulation. Proc Natl Acad Sci U S A 102: 743–748. 15640356 10.1073/pnas.0407692102 PMC545548

[105] Mueller JL, Linklater JR, Ravi Ram K, Chapman T, Wolfner MF (2008) Targeted gene deletion and phenotypic analysis of the Drosophila melanogaster seminal fluid protease inhibitor Acp62F. Genetics 178: 1605–1614. 18245332 10.1534/genetics.107.083766 PMC2278106

[106] Murer V, Spetz JF, Hengst U, Altrogge LM, de Agostini A, et al (2001) Male fertility defects in mice lacking the serine protease inhibitor protease nexin-1. Proc Natl Acad Sci U S A 98: 3029–3033. 11248026 10.1073/pnas.051630698 PMC30601

[107] Stanfield GM, Villeneuve AM (2006) Regulation of sperm activation by SWM-1 is required for reproductive success of *C. elegans* males. Curr Biol 16: 252–263. 16461278 10.1016/j.cub.2005.12.041

[108] Smith JR, Stanfield GM (2011) TRY-5 is a sperm-activating protease in *Caenorhabditis elegans* seminal fluid. PLoS Genet 7: e1002375. 22125495 10.1371/journal.pgen.1002375 PMC3219595

[109] Walker MJ, Rylett CM, Keen JN, Audsley N, Sajid M, et al (2006) Proteomic identification of *Drosophila melanogaster* male accessory gland proteins, including a pro-cathepsin and a soluble gamma-glutamyl transpeptidase. Proteome Sci 4: 9. 16670001 10.1186/1477-5956-4-9 PMC1462989

[110] Reinhardt K, Naylor RA, Siva-Jothy MT (2009) Ejaculate components delay reproductive senescence while elevating female reproductive rate in an insect. Proc Natl Acad Sci U S A 106: 21743–21747. 19996174 10.1073/pnas.0905347106 PMC2799855

[111] Pilch B, Mann M (2006) Large-scale and high-confidence proteomic analysis of human seminal plasma. Genome Biol 7: R40. 16709260 10.1186/gb-2006-7-5-r40 PMC1779515

[112] Dapples CC, Foster WA, Lea AO (1974) Ultrastructure of the accessory gland of the male mosquito, *Aedes aegypti* (L.) (Diptera: Culicidae). Int J Insect Morphol Embryol 3: 279–291.

[113] Ramalingam S (1983) Secretion in the male accessory-glands of *Aedes aegypti* (L.) (Diptera, Culicidae). Int J Insect Morphol Embryol 12: 87–96.

[114] Jones JC, Wheeler RE (1965) Studies on spermathecal filling in *Aedes aegypti* (Linnaeus). I. Description. Biol Bull 129: 134–150. 10.2307/1539731 5849123

[115] Marchini D, Del Bene G (2006) Comparative fine structural analysis of the male reproductive accessory glands in *Bactrocera oleae* and *Ceratitis capitata* (Diptera, Tephritidae). Ital J Zool 73: 15–25.

[116] Walters JR, Harrison RG (2008) EST analysis of male accessory glands from *Heliconius* butterflies with divergent mating systems. BMC Genomics 9: 592. 19063743 10.1186/1471-2164-9-592 PMC2621208

[117] Sirot LK, LaFlamme BA, Sitnik JL, Rubinstein CD, Avila FW, et al (2009) Molecular social interactions: *Drosophila melanogaster* seminal fluid proteins as a case study. Adv Genet 68: 23–56. 20109658 10.1016/S0065-2660(09)68002-0 PMC3925388

[118] Kovalick GE, Griffin DL (2005) Characterization of the SCP/TAPS gene family in *Drosophila melanogaster* . Insect Biochem Mol Biol 35: 825–835. 15944079 10.1016/j.ibmb.2005.03.003

[119] Roberts KP, Johnston DS, Nolan MA, Wooters JL, Waxmonsky NC, et al (2007) Structure and function of epididymal protein cysteine-rich secretory protein-1. Asian J Androl 9: 508–514. 17589788 10.1111/j.1745-7262.2007.00318.x

[120] LaFlamme BA, Ram KR, Wolfner MF (2012) The Drosophila melanogaster seminal fluid protease “seminase” regulates proteolytic and post-mating reproductive processes. PLoS Genet 8: e1002435. 22253601 10.1371/journal.pgen.1002435 PMC3257295

[121] Gibbs GM, Roelants K, O'Bryan MK (2008) The CAP superfamily: cysteine-rich secretory proteins, antigen 5, and pathogenesis-related 1 proteins–roles in reproduction, cancer, and immune defense. Endocr Rev 29: 865–897. 18824526 10.1210/er.2008-0032

[122] King TP, Spangfort MD (2000) Structure and biology of stinging insect venom allergens. Int Arch Allergy Immunol 123: 99–106. 11060481 10.1159/000024440

[123] Schreiber MC, Karlo JC, Kovalick GE (1997) A novel cDNA from *Drosophila* encoding a protein with similarity to mammalian cysteine-rich secretory proteins, wasp venom antigen 5, and plant group 1 pathogenesis-related proteins. Gene 191: 135–141. 9218711 10.1016/s0378-1119(97)00010-3

[124] Charlab R, Valenzuela JG, Rowton ED, Ribeiro JM (1999) Toward an understanding of the biochemical and pharmacological complexity of the saliva of a hematophagous sand fly *Lutzomyia longipalpis* . Proc Natl Acad Sci U S A 96: 15155–15160. 10611354 10.1073/pnas.96.26.15155 PMC24789

[125] Ameri M, Wang X, Wilkerson MJ, Kanost MR, Broce AB (2008) An immunoglobulin binding protein (antigen 5) of the stable fly (Diptera: Muscidae) salivary gland stimulates bovine immune responses. J Med Entomol 45: 94–101. 18283948 10.1603/0022-2585(2008)45[94:aibpao]2.0.co;2 PMC2580071

[126] Valenzuela JG, Pham VM, Garfield MK, Francischetti IM, Ribeiro JM (2002) Toward a description of the sialome of the adult female mosquito *Aedes aegypti* . Insect Biochem Mol Biol 32: 1101–1122. 12213246 10.1016/s0965-1748(02)00047-4

[127] Syed ZA, Härd T, Uv A, van Dijk-Härd IF (2008) A potential role for *Drosophila* mucins in development and physiology. PLoS One 3: e3041. 18725942 10.1371/journal.pone.0003041 PMC2515642

[128] Orr AG, Rutowski R (1991) The function of the sphragis in *Cressida cressida* (Fab.) (Lepidoptera, Papilionidae): a visual deterrent to copulation attempts. J Natural Hist 25: 703–710.

[129] Lung O, Wolfner MF (2001) Identification and characterization of the major Drosophila melanogaster mating plug protein. Insect Biochem Mol Biol 31: 543–551. 11267893 10.1016/s0965-1748(00)00154-5

[130] Moreira PL, Lopez L, Martin J (2006) Femoral secretions and copulatory plugs convey chemical information about male identity and dominance status in Iberian rock lizards (*Lacerta monticola*). Behav Ecol Sociobiol 60: 166–174.

[131] Rogers DW, Baldini F, Battaglia F, Panico M, Dell A, et al (2009) Transglutaminase-mediated semen coagulation controls sperm storage in the malaria mosquito. PLoS Biol 7: e1000272. 20027206 10.1371/journal.pbio.1000272 PMC2785878

[132] Wesseling J, van der Valk SW, Vos HL, Sonnenberg A, Hilkens J (1995) Episialin (MUC1) overexpression inhibits integrin-mediated cell adhesion to extracellular matrix components. J Cell Biol 129: 255–265. 7698991 10.1083/jcb.129.1.255 PMC2120361

[133] Chaturvedi P, Singh AP, Batra SK (2008) Structure, evolution, and biology of the MUC4 mucin. FASEB J 22: 966–981. 18024835 10.1096/fj.07-9673rev PMC2835492

[134] Baer B, Schmid-Hempel P (2001) Unexpected consequences of polyandry for parasitism and fitness in the bumblebee, *Bombus terrestris* . Evolution 55: 1639–1643. 11580023 10.1111/j.0014-3820.2001.tb00683.x

[135] Rawlings ND, Barrett AJ, Bateman A (2012) MEROPS: the database of proteolytic enzymes, their substrates and inhibitors. Nucleic Acids Res 40: D343–350. 22086950 10.1093/nar/gkr987 PMC3245014

[136] Dorus S, Busby SA, Gerike U, Shabanowitz J, Hunt DF, et al (2006) Genomic and functional evolution of the *Drosophila melanogaster* sperm proteome. Nat Genet 38: 1440–1445. 17099714 10.1038/ng1915

[137] Wasbrough ER, Dorus S, Hester S, Howard-Murkin J, Lilley K, et al (2010) The *Drosophila melanogaster* sperm proteome-II (DmSP-II). J Proteomics 73: 2171–2185. 20833280 10.1016/j.jprot.2010.09.002

[138] Dorus S, Wilkin EC, Karr TL (2011) Expansion and functional diversification of a leucyl aminopeptidase family that encodes the major protein constituents of *Drosophila* sperm. BMC Genomics 12: 177. 21466698 10.1186/1471-2164-12-177 PMC3078892

[139] Rosetto M, Belardinelli M, Fausto AM, Marchini D, Bongiorno G, et al (2003) A mammalian-like lipase gene is expressed in the female reproductive accessory glands of the sand fly *Phlebotomus papatasi* (Diptera, Psychodidae). Insect Mol Biol 12: 501–508. 12974955 10.1046/j.1365-2583.2003.00436.x

[140] Meikle D, Sheehan K, Phillis D, Richmond R (1990) Localization and longevity of seminal-fluid esterase-6 in mated female *Drosophila melanogaster* . J Insect Physiol 36: 93–101.

[141] Murphy EV, Zhang Y, Zhu W, Biggs J (1995) The human glioma pathogenesis-related protein is structurally related to plant pathogenesis-related proteins and its gene is expressed specifically in brain tumors. Gene 159: 131–135. 7607567 10.1016/0378-1119(95)00061-a

[142] Chen G, Gingerich J, Soper L, Douglas GR, White PA (2010) Induction of *lacZ* mutations in MutaMouse primary hepatocytes. Environ Mol Mutagen 51: 330–337. 19953605 10.1002/em.20540 PMC2959491

[143] Haynes SR, Cooper MT, Pype S, Stolow DT (1997) Involvement of a tissue-specific RNA recognition motif protein in *Drosophila* spermatogenesis. Mol Cell Biol 17: 2708–2715. 9111341 10.1128/mcb.17.5.2708 PMC232121

[144] Leopold R (1976) Role of male accessory glands in insect reproduction. Annu Rev Entomol 21: 199–221.

[145] Chen P (1984) The functional morphology and biochemistry of insect male accessory glands and their secretions. Annu Rev Entomol 29: 233–255.

[146] Couche G, Gillott C, Tobe S, Feyereisen R (1985) Juvenile hormone biosynthesis during sexual maturation and after mating in the adult migratory grasshopper, *Melanoplus sanguinipes* . Can J Zool 63: 2789–2792.

[147] Davey K (1985) The male reproductive tract. In: Kerket GA GL, editor. Comprehensive Insect Physiology, Biochemistry, and Pharmacology. Oxford: Pergamon. pp. 1–14.

[148] Regis L, Gomes Y, Furtado A (1985) Factors influencing male accessory-gland activity and 1st mating in *Triatoma infestans* and *Panstrongylus megistus* (Hemiptera, Reduviidae). Insect Science and Its Application 6: 579–583.

[149] Gold S, Davey K (1989) The effect of juvenile hormone on protein synthesis in the transparent accessory gland of male *Rhodnius prolixus* . Insect Biochem 19: 139–143.

[150] Gillott C (1988) Arthropoda-Insecta. In: Adiyodi R, Adiyodi K, editors. Reproductive biology of invertebrates. New York: Wiley and Sons. pp. 319–471.

[151] Wolfner MF, Partridge L, Lewin S, Kalb JM, Chapman T, et al (1997) Mating and hormonal triggers regulate accessory gland gene expression in male *Drosophila* . J Insect Physiol 43: 1117–1123. 12770484 10.1016/s0022-1910(97)00062-0

[152] Yamamoto K, Chadarevian A, Pellegrini M (1988) Juvenile hormone action mediated in male accessory glands of Drosophila by calcium and kinase C. Science 239: 916–919. 3124270 10.1126/science.3124270

[153] Wilson TG, DeMoor S, Lei J (2003) Juvenile hormone involvement in Drosophila melanogaster male reproduction as suggested by the Methoprene-tolerant(27) mutant phenotype. Insect Biochem Mol Biol 33: 1167–1175. 14599489 10.1016/j.ibmb.2003.06.007

[154] Tobe SS, Stay B (1985) Structure and regulation of the *corpus allatum* . Adv Insect Physiol 18: 305–432.

[155] Borovsky D, Carlson DA, Hancock RG, Rembold H, van Handel E (1994) De novo biosynthesis of juvenile hormone III and I by the accessory glands of the male mosquito. Insect Biochem Mol Biol 24: 437–444. 8205141 10.1016/0965-1748(94)90038-8

[156] Vannini L, Ciolfi S, Dallai R, Frati F, Hoffmann KH, et al (2010) Putative-farnesoic acid O-methyltransferase (FAMeT) in medfly reproduction. Arch Insect Biochem Physiol 75: 92–106. 20824822 10.1002/arch.20382

[157] Teal PE, Gomez-Simuta Y, Proveaux AT (2000) Mating experience and juvenile hormone enhance sexual signaling and mating in male Caribbean fruit flies. Proc Natl Acad Sci U S A 97: 3708–3712. 10706642 10.1073/pnas.060034397 PMC16304

[158] Zalewska M, Ożyhar A, Kochman M (2011) Identification of specific interaction of juvenile hormone binding protein with isocitrate dehydrogenase. Acta Biochim Pol 58: 119–124. 21403916

[159] Trowell SC (1992) High affinity juvenile hormone carrier proteins in the haemolymph of insects. Comp Biochem Physiol (B) 103: 795–807.

[160] De Kort CAD, Granger NA (1996) Regulation of JH titers: the relevance of degradative enzymes and binding proteins. Arch Insect Biochem Physiol 33: 1–26.

[161] Goodman WG, Granger NA (2005) The juvenile hormones. In: Gilbert LI, Iatrou K, Gill SS, editors. ScienceComprehensive Molecular Insect Science. Oxford: Elsevier. pp. 319–408.

[162] Fischer BE, Wasbrough E, Meadows LA, Randlet O, Dorus S, et al (2012) Conserved properties of Drosophila and human spermatozoal mRNA repertoires. Proc Biol Sci 279: 2636–2644. 22378807 10.1098/rspb.2012.0153 PMC3350705

[163] Simmerl E, Schäfer M, Schäfer U (1995) Structure and regulation of a gene cluster for male accessory gland transcripts in Drosophila melanogaster. Insect Biochem Mol Biol 25: 127–137. 7711745 10.1016/0965-1748(94)00034-f

[164] Cho KS, Won DH, Cha GH, Lee CC (2000) Regulation of Mst57Dc expression in male accessory glands of *Drosophila melanogaster* . Mol Cells 10: 180–185. 10850659 10.1007/s10059-000-0180-8

[165] Ellis LL, Carney GE (2010) Mating alters gene expression patterns in *Drosophila melanogaster* male heads. BMC Genomics 11: 558. 20937114 10.1186/1471-2164-11-558 PMC3091707

[166] Campbell PM, Healy MJ, Oakeshott JG (1992) Characterization of juvenile hormone esterase in *Drosophila melanogaster* . Insect Biochem Mol Biol 22: 665–677. 10.1016/s0965-1748(98)00037-x 9718682

[167] Campbell PM, Oakeshott JG, Healy MJ (1998) Purification and kinetic characterisation of juvenile hormone esterase from *Drosophila melanogaster* . Insect Biochem Mol Biol 28: 501–515. 9718682 10.1016/s0965-1748(98)00037-x

[168] Marchini D, Del Bene G, Falso LF, Dallai R (2001) Structural organization of the copulation site in the medfly *Ceratitis capitata* (Diptera: Tephritidae) and observations on sperm transfer and storage. Arth Struct & Dev 30: 39–54. 10.1016/s1467-8039(01)00018-4 18088943

[169] Vera MT, Cladera JL, Calcagno G, Vilardi JC, McInnis DO, et al (2003) Remating of wild *Ceratitis capitata* (Diptera: Tephritidae) females in field cages. Ann Entomol Soc Am 96: 563–570.

[170] Parker GA (1998) Sperm competition and the evolution of ejaculates: towards a theory base In: Birkhead T.R. MAP, editor. Sperm competition and sexual selection London, England: Academic 3–54.

