# Supplementary figures and images for: Transcriptional Profiles of Mating-Responsive Genes from Testes and Male Accessory Glands of the Mediterranean Fruit Fly, Ceratitis capitata
Source: PLoS One. 2012 Oct 11;7(10):e46812. doi: 10.1371/journal.pone.0046812 (PMC3469604; doi:10.1371/journal.pone.0046812)

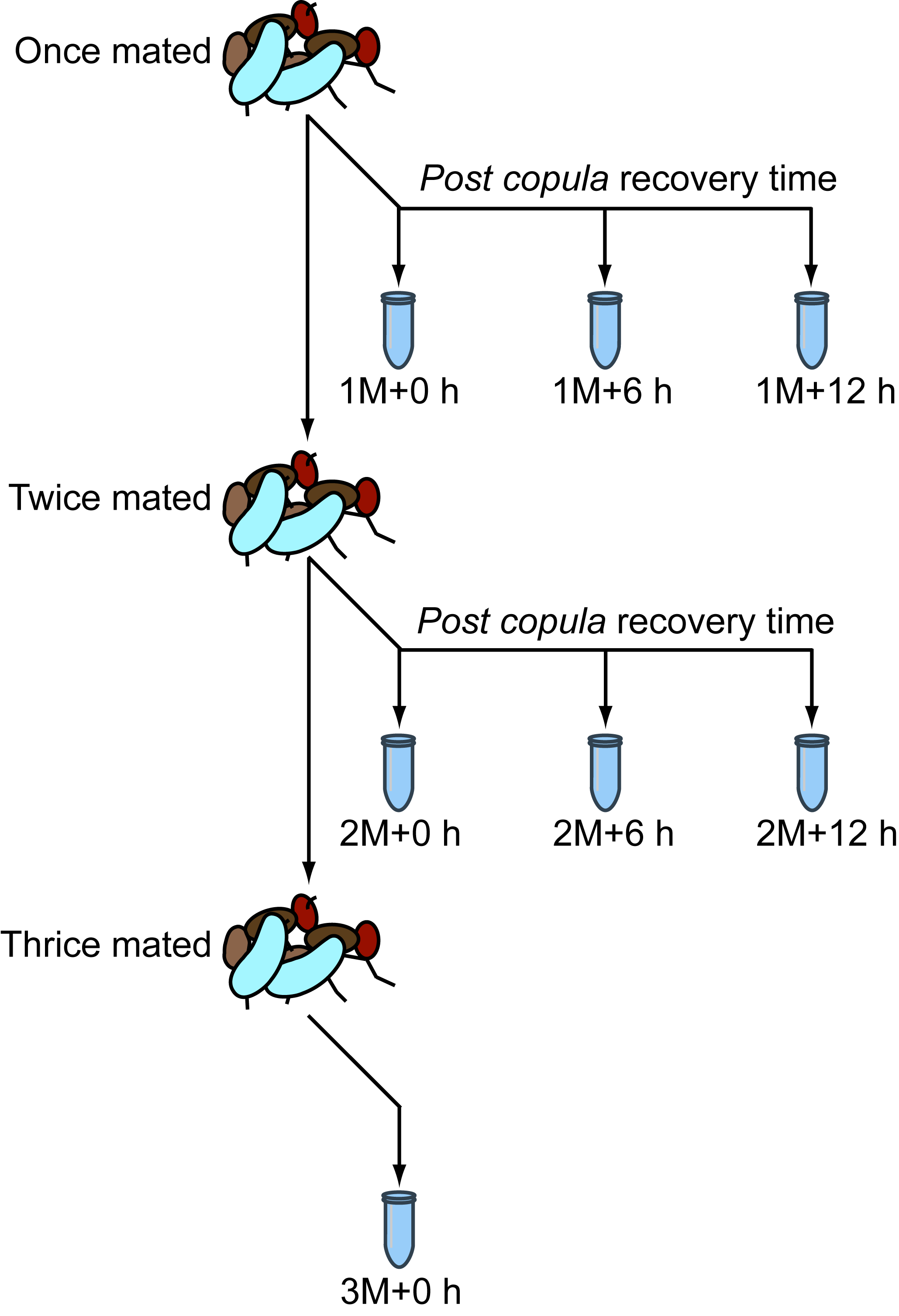

Supplement: Figure S1 — Schematic representation of the experimental design for Real-Time qPCR assays. Once mating was completed, individual males (once mated) were removed and analysed at designated recovery times for gene expression (0, 6 and 12 h). The remaining males that had mated were immediately allowed to remate (twice mated) and treated as above. Remaining twice mated males were immediately allowed to mate again (thrice mated) and all were sacrificed after the completion of the copula (0 h). At each time point, virgin males of the same age as the mated males were likewise sacrificed as controls. (TIF) [file pone.0046812.s001.tif]

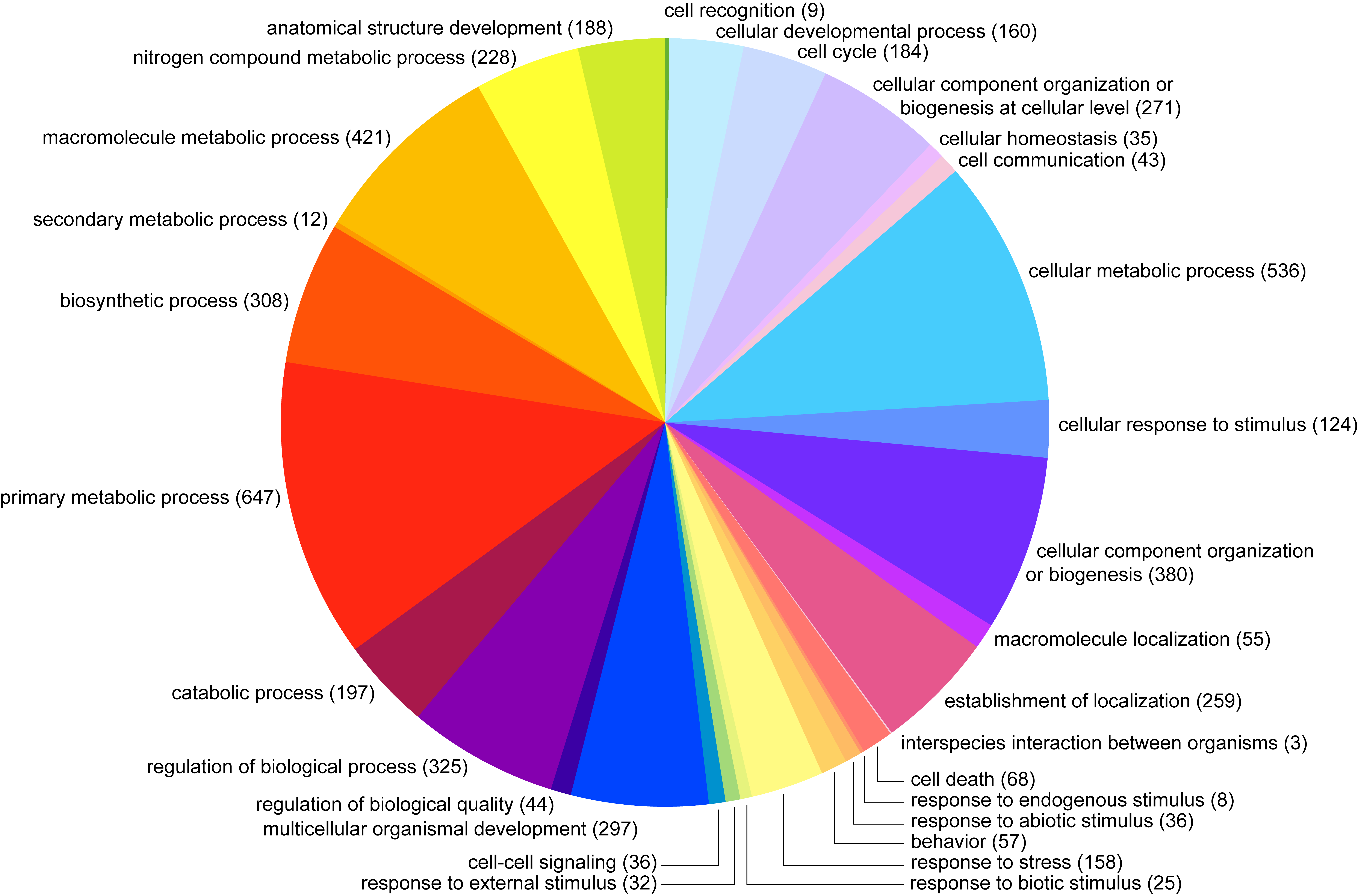

Supplement: Figure S2 — Distribution of the medfly TAG assembled sequences in Gene Ontology Biological Process categories Level III. (TIF) [file pone.0046812.s002.tif]

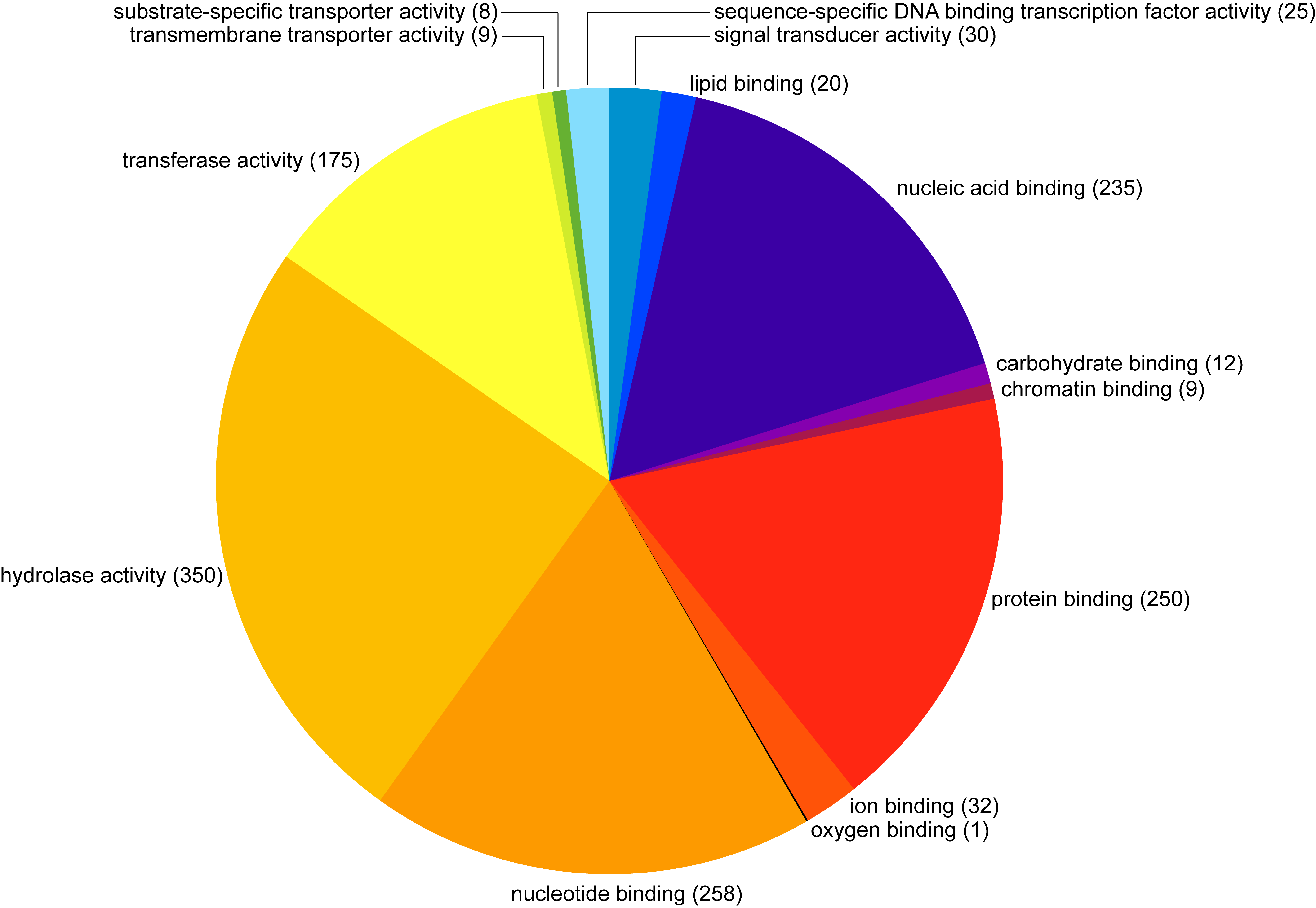

Supplement: Figure S3 — Distribution of the medfly TAG assembled sequences in Gene Ontology Molecular Function categories Level III. (TIF) [file pone.0046812.s003.tif]
