# Supplementary material for: Transcriptional Profiles of Mating-Responsive Genes from Testes and Male Accessory Glands of the Mediterranean Fruit Fly, Ceratitis capitata
Source: PLoS One. 2012 Oct 11;7(10):e46812. doi: 10.1371/journal.pone.0046812 (PMC3469604; doi:10.1371/journal.pone.0046812)
Supplement: Table S1 — Primers used in RT-PCR and Real-Time qPCR gene expression analyses. (DOC) [file pone.0046812.s004.doc]

Supplementary Table 1: Primers used in RT-PCR and Real-Time qPCR gene expression analyses. When possible, primers were designed to be ideal for RT-qPCR, with the melting temperature of the product close to 20°C above that of the primers, which were always designed to have an annealing temperature of 58°C

| **Primer name** | **Forward primer (5’ - 3’)** | **Reverse primer (5’ - 3’)** | **Expected product size** |
| --- | --- | --- | --- |
| TAG1006 | accagtttcgaaagcgaaga | gatagtcccggttagtggca | 203 |
| TAG1017 | attgtgtctgcccgtaaagg | agccatggaaatagcaccac | 137 |
| TAG1019 | acgcaatccttgtttttgtg | caccaccaccacctcctc | 143 |
| TAG1028 | tgcgatcatacctgaccaaa | tggtacacgctcaaattatctgtt | 189 |
| TAG1032 | gtgccgcattatccagagat | aagatttgaatttcgtgccg | 279 |
| TAG1041 | gtggtcaggttgacttccgt | accggatagttggcgtacag | 247 |
| TAG1050 | aatgtagcgaaagtaaagaggaaaa | gccaaactcttttgcagaca | 241 |
| TAG1090 | tcacagagcaacgaagatgg | caacggagccagtggtactt | 264 |
| TAG1105 | cgttcacgttcgtttcagtg | tgcgtcatctgtgccatatt | 174 |
| TAG1111 | atatgaacgaagcgttggagtt | ttcaccaattcctttcgttttt | 220 |
| TAG1117 | ggtgctagttttctggcgac | acccctgtgggaaatctctt | 219 |
| TAG1123 | acttgggcacgaaacgtaag | acgcttgatggtgatgttga | 122 |
| TAG1148 | gtatttttacgcgtttgggc | gccatatgacccaaagcaat | 285 |
| TAG1149 | gatcgctgagcagtgtttga | ctacctgccttagcgtggag | 187 |
| TAG1168 | gctgccacaaacccagtagt | agccgtcttgctgactgaat | 216 |
| TAG1221 | cgctgatgtcaaagcgtcta | tgtgtgaacgtacgcaaatg | 183 |
| TAG126 | ctggtgcgtacggtttatcc | gcatgtttccacaaacgaaa | 154 |
| TAG1261 | tcattctcactgcagaagcg | tttacagctgctggtgttgc | 270 |
| TAG1262 | ccgatgatgaatggactgtg | gcaactacctcagcctcgtc | 252 |
| TAG1282 | atgtgcaacaaacgatggtg | cgcaacctgttgctgttaat | 121 |
| TAG1290 | cattgcctattggtcgtgtg | ctcctgctgagggtgaactc | 272 |
| TAG1298 | ctcttggatcccaccgataa | tcagcactccaacacacctc | 158 |
| TAG1307 | aagatggcgctaagtgcttc | ctcatcagcggagaatgtca | 163 |
| TAG1341 | atacaagaacgatttggcgg | ccaattgatttgcattgacg | 253 |
| TAG1378 | tcctgggtttttcaatgagc | gccccactctttttcctttt | 99 |
| TAG1409 | aattcccgtcctgctgtatg | ctcactgttggaaactcgca | 107 |
| TAG1422 | tactggcgttatcggtcaca | cttgttgccatcttcggttt | 281 |
| TAG1431 | taccttcagccggcattatc | tcgcgttgatacaaaactcg | 135 |
| TAG1438 | cacataacactgccggtgag | tgcgtgaagaagtccaacag | 276 |
| TAG1448 | tgggattgcagctgtacaaa | acgtctcttacgcaccgttt | 162 |
| TAG146 | ttcttccgtaacggtgttcc | gaaccatcctttgcctcgta | 286 |
| TAG1461 | tgcgtgtgttcctgtgttta | tatgcagcaagccggaaac | 292 |
| TAG1468 | aatccatttttgcctgaacg | attccatggtggaaggacaa | 123 |
| TAG1478 | tgacagctgttcgttccttg | tgtctgcgttgaaggacttg | 113 |
| TAG148 | ggagcttctttcttggcctt | gtggacctcgaccagttgtt | 145 |
| TAG1487 | cggctgtcttttgatgttca | ttaatagtcactgccgccac | 198 |
| TAG1495 | ttaatcggcgctcttctgtt | tcgagcattgcagttgattt | 110 |
| TAG1500 | caagatgatcccaccctcat | accacccactaaatcggaca | 296 |
| TAG1511 | ttcgacatctcttggcttca | acaactcaaaggagccaacc | 203 |
| TAG1521 | ttgcttgtgatttgtgccat | cagctgatgagacgtcggta | 141 |
| TAG1523 | aaatagtcaacgcacacaatgc | aaactgatccatgccaaattct | 211 |
| TAG1524 | acaagaatattcgcgttggc | tacagtttgatgctgcctcg | 149 |
| TAG1529 | tggctacggtgttgttgtca | ggcatctctgaaggcatgtt | 96 |
| TAG1530 | tcgttcatgaaatccccttc | taaactcgtccaggcacctc | 113 |
| TAG1555 | catgctgtttctacttcggttg | atttccgcaaatcttcagtgtt | 151 |
| TAG1560 | atgtataacacgcggtccgt | taacgacatggattcggaca | 116 |
| TAG1563 | cgagcaaaagcaaaaggttc | cacagtcatctgcgccttta | 277 |
| TAG1570 | atgtcctcgcccacttatcc | gagccaagctcaggcaatag | 207 |
| TAG1572 | tacgcagagaatgccacaag | ctcgccatggaaggtatcat | 157 |
| TAG1577 | tgtaatcctggcagctgttg | cggtcgccattgaagttatt | 239 |
| TAG1580 | tacgcagagaatgccacaag | tcattcttatctgcgcaacg | 237 |
| TAG1602 | cacataaccatgacaaggcg | tggtgtagctcagatgcctg | 206 |
| TAG1613 | cagtcaacatttccgaattcaa | gtgcagttaatgagcactctcg | 217 |
| TAG162 | tacctcgttgcattgctctg | ttttctccttgtggtcggtc | 207 |
| TAG1629 | catcacgaacaccattccag | atcatcgccgttcagaaaac | 259 |
| TAG1668 | cattgcttgcattagcgaaa | gtgcagacgaacattgtgct | 203 |
| TAG1671 | ctcggatgtcgtggaagaat | ttggcttcacagtcctcctt | 167 |
| TAG1672 | aagaatgccatcaacccaag | tcaactacaggcaccacagg | 264 |
| TAG1692 | acaacgagcgcgtctattct | gtggaactaccagttgcggt | 238 |
| TAG1693 | gttgagcttgtcgaatgcaa | accaccaatcatttgggaaa | 126 |
| TAG1695 | acaacgagcgcgtctattct | gtggaactaccagttgcggt | 238 |
| TAG173 | aaacattggaactccgcaac | gttatgcccaacgctagagg | 108 |
| TAG1741 | tgaaccagtgcaaacgtgat | aaagaatcggtcgcagaaga | 139 |
| TAG1742 | ggcacaagttcatacagcca | cctttacactcgaacgggaa | 123 |
| TAG1799 | ttcgccatcgttatttcctc | caccgttgtgattttcgatg | 197 |
| TAG1822 | ttggggaagatcccctttt | cggggcatattttacattgg | 116 |
| TAG1836 | gtatcgatggcgcgtttagt | atcaatccaatagccccaca | 144 |
| TAG1848 | gttatgtccggtggagcact | caccgttcacattatggctg | 156 |
| TAG1874 | gttacgttattgctgggcgt | ccgccatgatatgccttact | 265 |
| TAG1899 | gggaggacttctttttgggt | taaaccggcctcttttccac | 125 |
| TAG1902 | tcacattccatttggcaaca | acgagacgagagaaaaagcg | 133 |
| TAG1989 | tatttggtgtatgctgccca | tttgctttagctgccacctt | 128 |
| TAG1997 | aagaatgggtggttccacaa | tgcccctgtttggtgtattt | 175 |
| TAG1999 | cctgtcccaaaggggtttat | ttaacccggaaaaacactcg | 184 |
| TAG2013 | ccagatccactgtacgggtt | caaacttggagggaaaccaa | 164 |
| TAG2014 | gacaaaacaagggaccgaaa | ctgtttccgtctgttgagca | 176 |
| TAG2032 | atgtgtgcagtcgtgttggt | ctctccgtttgttggttggt | 181 |
| TAG207 | ccactgaacgatttccgttt | ttctgacacaaagcagcacc | 249 |
| TAG208 | cgaattggtcattcgtcctt | ggccatggtattgtaatcgg | 199 |
| TAG2095 | cctcgctggactcttttacg | ttattgccgacaaatgcaaa | 267 |
| TAG2111 | cgttgtaggcaatgacgaga | cgagcaaactgaagcaacaa | 154 |
| TAG2130 | tggcaccatctgacattttt | ccaaaaagttgttgtaggcga | 112 |
| TAG2142 | gcctcgtgccgatacatac | cgaaaacaattgatgaaagctg | 156 |
| TAG2169 | ttgcccactacattgcaaaa | tttgctctcagcaaccaatg | 173 |
| TAG2170 | aggcgaacataagcctttca | tccaagtgttcctcgtcgat | 178 |
| TAG2172 | cttccggattggtgaagcta | ctatccaccgcttccaaaaa | 130 |
| TAG2176 | ccttttctccttcctttcgg | taaaacctttttccaggggg | 231 |
| TAG221 | acgaatccattccataccca | ggcaaactcgttttgctctc | 153 |
| TAG2241 | tcgccttcaaaatatgctcc | tttttgagttcgcagtggg | 105 |
| TAG2253 | cttcggtgtagctgatgcaa | ccaaatgcagagtctcacga | 181 |
| TAG2254 | gccgatactttggatcagga | actcgcagattttcgcatct | 231 |
| TAG2256 | cttcctccgaatcctcagtg | tatacgtggcgacggtgtta | 163 |
| TAG228 | ttgacatttgctgtaattttattattg | aaaagcacagaatcgtttgaaaat | 89 |
| TAG2283 | tctaggattcggcggtattg | gtaaccgaagcgcgagtaag | 228 |
| TAG2291 | ccctatttgccttttcctcc | ctgaatgtgggcggaatatc | 249 |
| TAG230 | caagcaccgctagttgtgaa | gcaatagcacctcctgcttc | 192 |
| TAG2301 | tgcaacagctacaagcctca | agcggagagagaacactcca | 155 |
| TAG2314 | cggttccaggatgacacttt | tctcagctttggcctttgtt | 249 |
| TAG2321 | tgggaaattcactagggcac | ccgggcactcactaaacatt | 186 |
| TAG2322 | tttgggcctcatacttttgg | aaattcaaactgcgtcctcg | 182 |
| TAG2332 | aaaacgttgcagaaatcgct | gcagtgcgtgttactgccta | 236 |
| TAG2346 | tgcttttcttcctcgctttt | gccaagcagagtttgtttcc | 102 |
| TAG2356 | gaccgtgtgcaccagttgta | ctggagacgggtatgtgacc | 240 |
| TAG2368 | acagctatcgcgctcttctc | gtcggacttcctgaccacat | 134 |
| TAG2399 | tcctgtacaaacaacgacgc | ttgcacagctttagtccacg | 123 |
| TAG2405 | ttgatagctgttggcgctta | gtttgagtcggttcgtctgc | 172 |
| TAG2417 | acaccaacccaaatctctgc | gagctgtacgacagcgatga | 286 |
| TAG2424 | taataaacccccttcctgcc | gggtttataaaaattttggggg | 153 |
| TAG2430 | gttgcggtgctgagtctaca | tgtccatagcgtgcagtttc | 276 |
| TAG2443 | ttgccttttgtgtttacccc | tgggcgtataaccctttgag | 194 |
| TAG2458 | agaggagatgctgcgtcaat | cgcagtgcatcttctcacat | 157 |
| TAG2481 | cgctttacctcctctattcgg | tggaatgttttggttgacga | 137 |
| TAG250 | atattgtggcaagcctttgg | gcctgcgtgtttaatggttt | 157 |
| TAG2502 | cggcaaatacaatgtgatcg | catgagcgcctaaactgtga | 181 |
| TAG2529 | tttgtgagcttcttgttcgg | ttgggaatgaatgaacccat | 212 |
| TAG2538 | agttttcgcttccatgctgt | ccaagatgcgagtgctacaa | 171 |
| TAG2558 | acatttgcaacagccagtga | tattgcaatgctcacctcca | 144 |
| TAG2568 | tcaggctgcacagaatcaac | gcataacggtttggcgtact | 131 |
| TAG2570 | agatccaagttgggcgtatg | ttctgctgaaggcataggaaa | 111 |
| TAG258 | tgtgtgggctttgcgtatta | cgtaatatgagctcgcttgc | 195 |
| TAG2602 | aaggcattcaaaccgacatc | tggctgaacgctacagaatg | 156 |
| TAG2607 | tggcgaaagtcaactcagtg | gtctgctagccgagttcacc | 130 |
| TAG2627 | gtcttcgctatcttcgctgg | ttggactgtggcatctcttg | 101 |
| TAG2680 | tcgaaatcacgttattctgtca | cgacgagcatttcaaaatca | 125 |
| TAG2693 | tgattatgttatttaaagggaggca | aaaaggggcataggagctgt | 94 |
| TAG2733 | gactttgcgttgtgctgtgt | gctgttttagcggtttctgc | 216 |
| TAG2748 | gtggatatacaaatggcggc | agccagtttttcatgctgct | 199 |
| TAG276 | cgttgctgagttgagtcgtc | caaacacacacagcctgtcc | 144 |
| TAG2769 | caaattaaagccgtgggaga | gcgttaatggtgtgctctca | 112 |
| TAG277 | aacgtctactgcctagccga | tgccgacaaagtaactgctg | 144 |
| TAG2779 | agtgctcattgtgttcgctg | gagcaagaattggtgcatga | 157 |
| TAG2792 | actttgaggaagcgaaaacg | tgcggcataacggtacaata | 246 |
| TAG280 | agcagctcgtgatgtttgtg | agatcttgttcgcgctttgt | 227 |
| TAG2810 | taaattgggcagctttgctt | cgcagcatttgaatcgtcta | 118 |
| TAG2821 | ctcgctcatagttgcgatca | gtttgtcgggtccgctagta | 171 |
| TAG2832 | cttgcaccattggactcaac | atccagtccagccaaagaaa | 147 |
| TAG2849 | ttttctgcttttatgccgct | ctgcgaaaaacaaagactcg | 159 |
| TAG2856 | ccgtcttcactttggctctc | tgagcaacataagcaccacc | 267 |
| TAG2860 | ggttcggtggtgaagagtgt | agtttggtgaacaaccggag | 221 |
| TAG2889 | tttaattgttattgttgttgttgcat | taccgccaaacagcagaac | 91 |
| TAG289 | tgtcgctaatgcagaacagg | ctcgcattttgtttgcttca | 193 |
| TAG2890 | ctcagccgatggtaatggtt | attcttattgccgccacaac | 117 |
| TAG2907 | ggctcttgctgtagtccctg | ttattggcactcgaaatccc | 186 |
| TAG2911 | gacagtggatccgatgaggt | gccgaagattgaagagcaac | 109 |
| TAG2913 | tccggtgtaccatcatcaga | cgaggaccaagatgaggaaa | 114 |
| TAG2926 | ctttgtgataccgctcgctt | gcggttaaagccatgcttac | 170 |
| TAG2960 | aaggattcgtgttctggtgatt | tcgccttaatcctttcaaacat | 197 |
| TAG2963 | cataaatgcaacacggatcg | aatacgagttggttgcggtc | 150 |
| TAG2983 | tagttgcgacagcattgtcc | gtcccaatcgtgttagtggg | 173 |
| TAG299 | ttcttttcgctcttcaccgt | tagagccgcagatttgtcct | 111 |
| TAG3020 | tgttaactgcagcgacttgc | cttcagttcattgccccatt | 130 |
| TAG3021 | ttggattccagtgcagcata | taaccatgggataatcggga | 150 |
| TAG3024 | cactctgataccgccgattt | tcattagcctgctccgactt | 136 |
| TAG3030 | ccaggtattaaaccagcgga | aggtgactgtccaccacctc | 227 |
| TAG3102 | cgggtacgcaacatcacata | ttgcaatcggaacgtaatga | 171 |
| TAG3137 | aagtgggtgcaaaagaggtcta | ataaccaagtagcaacggcatt | 155 |
| TAG3143 | acgctcgatcaaaatatcgg | cagaataaacgacgcgcata | 289 |
| TAG315 | gccatgaagcattcgatttt | gcagcaaccaaccgtatttt | 149 |
| TAG3150 | tatggacatgcttgctacgg | tcaacaggtcgcttcagatg | 241 |
| TAG3175 | tcaccatgtatgttttcttgtttg | tcataaaagtgctgagtgcaa | 133 |
| TAG3206 | tgggcctttgtgtttctacc | ccgaggacgttttcaatgat | 253 |
| TAG3250 | gaagtgattgatgtgccacg | ggtattgcctttggagggat | 284 |
| TAG3261 | catggtgtacggcacaaaag | gcacaactccttgtcccatt | 248 |
| TAG3266 | ttttccctgttcgtcgtctt | actcaaatccaggccatcac | 107 |
| TAG3272 | tgcggaattaaagggtgaac | accttccaagatggcatcac | 165 |
| TAG3289 | tcgacacaacattcaccgat | gtcaggacgaaggtgagcat | 252 |
| TAG3291 | ttcggtattggtgcttttcc | tgcgaacaataaccaagcaa | 94 |
| TAG330 | gccttgtgccttacttttgc | ttacgactttctggcacacg | 113 |
| TAG3320 | cggtcggtgataaaccaact | tggcttacaaacatgccaaa | 213 |
| TAG3324 | acgccgctagactgatgttt | tattcgatccgattggcttc | 191 |
| TAG359 | aattggcattcttaatggcg | taaccaccctgagcacatga | 265 |
| TAG368 | aaaagacggatgcataaccg | tgcagcttctgctgctttag | 299 |
| TAG37 | gacgaggtgcgtcgtctaat | ataatgcaattgctgcacga | 161 |
| TAG372 | gcctggtggatgtcactttt | cctagctccagatgaggcag | 128 |
| TAG389 | tgggtaaaaggaaatcagcg | tttagctgggcaaccaattc | 180 |
| TAG40 | gaaacagcgcgtcagtacaa | gttgatgtgagcaagctgga | 255 |
| TAG404 | agtttgaaatgtggcttgtgtg | aagtacgcttgtcgtccaaaat | 184 |
| TAG412 | cccttgttgggacctgatatt | aggtgggcgtatatccgttt | 118 |
| TAG420 | tgaagagatgaaaacgtgcg | gctaggatttcgcttgcatc | 125 |
| TAG421 | ggtattgcttgtgtcagcga | gcgacgaatcacaagtcaaa | 294 |
| TAG435 | tgaggctacatcagcactgg | atcgtcactttcgtcgctct | 270 |
| TAG443 | attgcccttcattggttttg | accaagaaggaggcttaccc | 130 |
| TAG458 | attgggttcacgcttaccag | tgacttccacgctcgttatg | 145 |
| TAG474 | tcgactttacgcccaatttc | ttgtgtagggattaagcggg | 119 |
| TAG489 | tcttactaaagcaggccaccat | gtagcgatggcacattatttca | 205 |
| TAG510 | atggcgagttggtaaaggtg | tgtcgctggtatcgtctctg | 154 |
| TAG534 | gcacgaggttggaatatgct | gctcttttcagtagtgggcg | 213 |
| TAG546 | gataagcccagcaaggatga | agcaggttgtgctggttctt | 167 |
| TAG552 | acttgggtgttgtcgtcctc | taaagcggtggctctcctaa | 247 |
| TAG588 | tatccctattggggctttcc | tattctgcaaattgggccag | 106 |
| TAG602 | accatcgccaaactttgaac | tcctcttgctccggatattg | 293 |
| TAG623 | acttatttggatcggtggattg | aaacacacgcgtacataagtgg | 162 |
| TAG704 | actttgtgacgtgtgggtca | cacattgtacgggttcacca | 200 |
| TAG707 | ggaacaattcaccgctttgt | gcgttcgggtgtgtagaaat | 177 |
| TAG711 | cccccaaccctttttgttat | gtggctgtaaataacccgga | 106 |
| TAG745 | attcccagtttccctctggt | gaactcgtcgccgaaaaata | 112 |
| TAG764 | gtggacactgaagaggagcc | ccttccaagaacgagcatgt | 139 |
| TAG77 | cgattcaactcaaattgcca | ccggcaagaacttcagaaag | 165 |
| TAG771 | aaatgaattcgacacgcaca | agtttggccaataccaccag | 280 |
| TAG791 | tacgcctgtttgtgcttctg | gaagagacaacgattgccgt | 282 |
| TAG792 | ttctgcaattatggtctgcg | ggagtgtccagagctccaaa | 145 |
| TAG836 | tgttgctgtagtggctttgc | tgtgtgtgttttcgtgctga | 103 |
| TAG839 | gctggctacgatatttggct | gtctcgtagagtcgcacacg | 123 |
| TAG850 | cacagcagccattgagtcat | tgttcactgtaaccaagccg | 138 |
| TAG857 | catttggatatacgtggtgtgg | gggttggtgtgaggaacatact | 177 |
| TAG863 | tccacgctaatcctggaaac | atgcaggattgctgctttct | 188 |
