# Supplementary material for: Transcriptional Profiles of Mating-Responsive Genes from Testes and Male Accessory Glands of the Mediterranean Fruit Fly, Ceratitis capitata
Source: PLoS One. 2012 Oct 11;7(10):e46812. doi: 10.1371/journal.pone.0046812 (PMC3469604; doi:10.1371/journal.pone.0046812)
Supplement: Table S2 — Descriptions of the 65 contigs derived from the most abundant transcripts in the medfly testes/male accessory glands transcriptome. (DOC) [file pone.0046812.s005.doc]

Supplementary Table 2: Descriptions of the 65 contigs derived from the most abundant transcripts in the medfly testes/male accessory glands transcriptome.

| **Contig** | **Reads** | **Accession no. of best hit** | **Description of best BLASTX or FASTY^a^ hit [species]** | **e-value** |
| --- | --- | --- | --- | --- |
| TAG1605 | 75 | CAM36311.1 | hypothetical protein [*Thermobia domestica*] | 8.0e-08 |
| TAG1572 | 53 | CAA70152.1 | male-specific protein [*Ceratitis capitata*] | 8.0e-88 |
| TAG1577 | 44 | CAB64647.1 | male specific serum polypeptide beta 2 [*Ceratitis capitata*] | 8.0e-83 |
| TAG1579 | 41 | CAB64647.1 | male specific serum polypeptide beta 2 [*Ceratitis capitata*] | 2.0e-85 |
| TAG1575 | 35 | CAB64651.1 | male specific serum polypeptide alpha 1 [*Ceratitis capitata*] | 5.0e-88 |
| TAG2931 | 31 | None |  |  |
| TAG2941 | 29 | ADD19015.1 | cyclophilin type peptidyl-prolyl cis-trans isomerase [*Glossina morsitans morsitans*] | 3.0e-109 |
| TAG158 | 28 | NP_524402.1 | ATP synthase, subunit d [*Drosophila melanogaster*] | 2.0e-86 |
| TAG1638 | 28 | NP_049374.1 | polyprotein [Sacbrood virus] | 7.0e-78 |
| TAG1574 | 25 | CAB64651.1 | male specific serum polypeptide alpha 1 [*Ceratitis capitata*] | 1.0e-86 |
| TAG1692 | 20 | None |  |  |
| TAG1587 | 18 | P04357.1 | Metallothionein A [*Drosophila melanogaster*]^a^ | 3.6e-08 |
| TAG1688 | 18 | None |  |  |
| TAG851 | 16 | NP_572610.1 | lethal (1) G0230 [*Drosophila melanogaster*] | 9.0e-60 |
| TAG1702 | 16 | None |  |  |
| TAG1578 | 15 | CAB64647.1 | male specific serum polypeptide beta 2 [*Ceratitis capitata*] | 1.0e-85 |
| TAG1778 | 15 | NP_573196.1 | CG5010 [*Drosophila melanogaster*] | 3.0e-32 |
| TAG402 | 15 | XP_001656475.1 | hypothetical protein AaeL_AAEL003177 [*Aedes aegypti*] | 7.0e-10 |
| TAG1588 | 14 | None |  |  |
| TAG875 | 13 | NP_725995.1 | exuperantia [*Drosophila melanogaster*] | 3.0e-161 |
| TAG1618 | 13 | XP_002054427.1 | GJ22814 [*Drosophila virilis*] | 5.0e-06 |
| TAG1639 | 13 | ADN38255.1 | polyprotein [Sacbrood virus CSBV-LN/China/2009] | 0.0 |
| TAG2981 | 13 | None |  |  |
| TAG2982 | 13 | None |  |  |
| TAG191 | 13 | NP_524358.2 | oligomycin sensitivity-conferring protein [*Drosophila melanogaster*] | 2.0e-100 |
| TAG430 | 12 | NP_524011.1 | ATP synthase, subunit b [*Drosophila melanogaster*] | 2.0e-141 |
| TAG449 | 12 | NP_731384.1 | spermidine synthase [*Drosophila melanogaster*] | 2.0e-154 |
| TAG1557 | 12 | None |  |  |
| TAG427 | 11 | NP_608747.2 | CG17261 [*Drosophila melanogaster*] | 5.0e-11 |
| TAG1563 | 11 | NP_611444.2 | Odorant-binding protein 56d [*Drosophila melanogaster*] | 5.0e-27 |
| TAG3257 | 11 | NP_728294.1 | CG14235, isoform A [*Drosophila melanogaster*] | 2.0e-45 |
| TAG693 | 10 | NP_524586.1 | myosin light chain 2 [*Drosophila melanogaster*] | 8.0e-86 |
| TAG1068 | 10 | NP_524728.2 | eukaryotic initiation factor 1A [*Drosophila melanogaster*] | 2.0e-63 |
| TAG1223 | 10 | None |  |  |
| TAG1252 | 10 | NP_610376.1 | CG8701 [*Drosophila melanogaster*] | 2.0e-46 |
| TAG1580 | 10 | CAB64650.1 | male specific serum polypeptide gamma 1 [*Ceratitis capitata*] | 4.0e-78 |
| TAG3254 | 10 | XP_001356918.2 | GA19266 [*Drosophila pseudoobscura pseudoobscura*] | 2.0e-09 |
| TAG507 | 9 | NP_524808.2 | elongation factor 1 beta [*Drosophila melanogaster*] | 2.0e-98 |
| TAG557 | 9 | NP_724357.1 | elongation factor 2b [*Drosophila melanogaster*] | 0.0 |
| TAG846 | 9 | CAA70286.1 | cytochrome c oxidase subunit Va preprotein [*Drosophila melanogaster*] | 1.0e-68 |
| TAG879 | 9 | NP_477034.1 | twinstar [*Drosophila melanogaster*] | 6.0e-103 |
| TAG1532 | 9 | None |  |  |
| TAG2898 | 9 | None |  |  |
| TAG25 | 9 | CAA32434.1 | H3 histone [*Drosophila melanogaster*] | 2.0e-86 |
| TAG181 | 8 | NP_608326.1 | HspB8 [*Drosophila melanogaster*] | 4.0e-105 |
| TAG436 | 8 | NP_996281.1 | CG33340 [*Drosophila melanogaster*] | 7.0e-10 |
| TAG640 | 8 | NP_476772.1 | alpha-Tubulin at 84B [*Drosophila melanogaster*] | 0.0 |
| TAG707 | 8 | CBA35213.1 | CG5210 protein [*Drosophila melanogaster*] | 0.0 |
| TAG868 | 8 | NP_476667.1 | polyA-binding protein [*Drosophila melanogaster*] | 4.0e-151 |
| TAG1003 | 8 | P26228.1 | protease inhibitor [*Sarcophaga bullata*] | 3.0e-28 |
| TAG1268 | 8 | NP_477375.1 | elongation factor 1alpha48D [*Drosophila melanogaster*] | 2.0e-160 |
| TAG1560 | 8 | None |  |  |
| TAG1565 | 8 | NP_611444.2 | Odorant-binding protein 56d [*Drosophila melanogaster*] | 5.0e-27 |
| TAG1581 | 8 | CAB64650.1 | male specific serum polypeptide gamma 1 [*Ceratitis capitata*] | 7.0e-79 |
| TAG1608 | 8 | NP_652413.1 | CG7630 [*Drosophila melanogaster*] | 7.0e-09 |
| TAG1662 | 8 | NP_610440.1 | CG13747 [*Drosophila melanogaster*] | 8.0e-23 |
| TAG1686 | 8 | NP_609839.2 | CG5050 [*Drosophila melanogaster*] | 3.0e-30 |
| TAG1694 | 8 | None |  |  |
| TAG1704 | 8 | None |  |  |
| TAG1863 | 8 | ABE68806.1 | cytochrome oxidase subunit III [*Ceratitis capitata*] | 2.0e-139 |
| TAG2607 | 8 | NP_572542.1 | CG15369 [*Drosophila melanogaster*] | 4.0e-22 |
| TAG2925 | 8 | NP_570056.1 | CG14269 [*Drosophila melanogaster*] | 4.0e-47 |
| TAG2954 | 8 | NP_651171.1 | CG10252 [*Drosophila melanogaster*] | 4.0e-90 |
| TAG3032 | 8 | None |  |  |
| TAG3302 | 8 | AAB26519.1 | glutathione S-transferase D1 [*Drosophila melanogaster*] | 4.0e-88 |
