# Supplementary material for: Transcriptional Profiles of Mating-Responsive Genes from Testes and Male Accessory Glands of the Mediterranean Fruit Fly, Ceratitis capitata
Source: PLoS One. 2012 Oct 11;7(10):e46812. doi: 10.1371/journal.pone.0046812 (PMC3469604; doi:10.1371/journal.pone.0046812)
Supplement: Table S3 — Classification of TAG peptides with putative housekeeping functions. (DOC) [file pone.0046812.s006.doc]

Supplementary Table 3: Classification of TAG peptides with putative housekeeping functions.

| **Class** | **Number of peptides** | **%** |
| --- | --- | --- |
| Unknown conserved | 596 | 27.59 |
| Protein synthesis | 174 | 8.06 |
| Protein modification machinery | 164 | 7.59 |
| Signal transduction | 156 | 7.22 |
| Metabolism, Energy | 152 | 7.04 |
| Transcription machinery | 130 | 6.02 |
| Transporters and storage | 111 | 5.14 |
| Cytoskeletal | 96 | 4.44 |
| Metabolism, Lipid | 89 | 4.12 |
| Metabolism, Carbohydrate | 66 | 3.06 |
| Protein export machinery | 66 | 3.06 |
| Nuclear regulation | 55 | 2.55 |
| Metabolism, Aminoacid | 45 | 2.08 |
| Transcription factor | 40 | 1.85 |
| Proteasome machinery | 39 | 1.81 |
| Nucleotide metabolism | 38 | 1.76 |
| Detoxification | 36 | 1.67 |
| Extracellular matrix and adhesion | 31 | 1.44 |
| Intermediary metabolism | 28 | 1.30 |
| Immunity | 17 | 0.79 |
| Oxidant metabolism/detoxication | 15 | 0.69 |
| Apoptosis | 13 | 0.60 |
| Nuclear export | 3 | 0.14 |
| Total | 2160 | 100 |
